# Comorbidity profiles in chronic obstructive pulmonary disease: a multicohort study

**DOI:** 10.1183/23120541.01289-2024

**Published:** 2025-10-27

**Authors:** Line Egerod, Else A.M.D. ter Haar, Morten A. Karsdal, Diana J. Leeming, Carmel B. Nanthakumar, Julie C. Yates, Dirk-Jan Slebos, Simon D. Pouwels, Jorine E. Hartman, Jannie M.B. Sand

**Affiliations:** 1 Department of Biomedical Sciences, University of Copenhagen, Copenhagen, Denmark; 2 Nordic Bioscience, Herlev, Denmark; 3 University of Groningen, University Medical Center Groningen, Groningen Research Institute for Asthma and COPD, Groningen, The Netherlands; 4 University of Groningen, University Medical Center Groningen, Department of Pulmonary Diseases, Groningen, The Netherlands; 5 Respiratory Biology, GSK-HQ, London, UK; 6 GSK R&D, Durham, NC, USA

## Abstract

**Background:**

Comorbidities are common in COPD, adversely affecting patients’ health. Cluster analysis has been proposed to study phenotypic variability, but inconsistent results have raised concerns. This study uses uniform machine learning techniques across large COPD cohorts to identify comorbidity clusters.

**Methods:**

Cluster analysis was conducted using data from COPD patients from the ECLIPSE study. 13 comorbidities were grouped using hierarchical clustering on self-organising maps to identify dominant subgroups. The analysis was replicated in COPD patients from the Groningen Severe COPD Cohort (GSCC).

**Results:**

2054 of 2164 ECLIPSE participants (1337 (65%) men; mean±sd age 63.3±7.1 years; 22.0% Global Initiative for Chronic Obstructive Lung Disease (GOLD) stage E) with sufficient data were included in the analysis. Four clusters were identified: Musculoskeletal (C1: more females, lower nutritional status), Mental (C2: younger females, highest comorbidity count), Circulatory (C3: more males, older) and Metabolic (C4: older males, high nutritional status). The replication analysis (776 of 1030 GSCC participants; 266 (34.3%) men; mean±sd age 61.4±6.9 years; 58.9% GOLD stage E) confirmed three clusters. Two clusters (C1, C2) showed similar phenotypic signatures to those in ECLIPSE, while the third combined features of C3 and C4. In ECLIPSE, C1 was associated with increased mortality (HR 2.30, 95% CI 1.26–4.13, p=0.006), which was not replicated in GSSC.

**Conclusion:**

Comorbidity clusters exist in COPD and are linked to different patient subgroups with varying symptom burden. While cohort-specific variations in disease outcomes were observed, overall, the study's findings support the presence of comorbidity profiles with potential to improve disease management.

## Shareable abstract

**Comorbidities in COPD group into profiles, identifying clusters of patients with varying disease burdens. These findings support more patient-centred strategies to potentially improve disease management.** https://bit.ly/4iIkkle

## GRAPHICAL ABSTRACT

**Figure:**
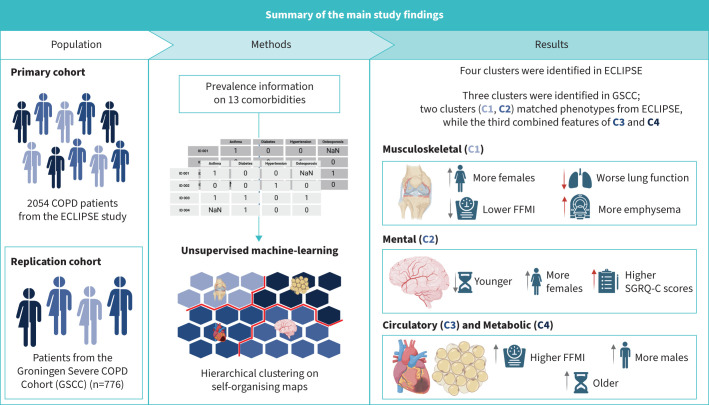
**Summary of the main study findings.** Clustering analyses across two large COPD cohorts show reproducible comorbidity clusters with varying clinical characteristics and symptom burdens. SGRQ-C: COPD-specific version of the St. George's Respiratory Questionnaire; FFMI: fat-free mass index.

## Introduction

COPD—a heterogeneous disorder characterised by airflow obstruction [1]—extends beyond respiratory issues. Most patients with COPD deal with multiple morbidities presenting a complex underlying comorbidity landscape [2, 3]. The most common chronic diseases associated with COPD include osteoporosis, muscle wasting, chronic kidney disease, gastro-oesophageal reflux, anaemia, cancer, and cardiovascular, metabolic and neuropsychiatric disorders [2–6]. These comorbidities exacerbate symptoms, hinder daily function, compromise overall well-being [7], and increase the risks of hospitalisation [8] and mortality [4, 8].

The Global Initiative for Chronic Obstructive Lung Disease (GOLD) guidelines recognise the importance of comorbidities in COPD but recommend a single-disease-oriented strategy by managing and treating individual diseases separately [6]. This strategy has limitations [9] and a risk of adverse effects from multiple drug prescriptions [10]. To advance patient care, it is crucial to identify subgroups of patients with similar observable profiles (*i.e.*, phenotypes) that may share underlying biological causes (*i.e.*, endotypes) [11]. As an alternative to current recommendations, using clusters of comorbidities could present a more integrated and patient-centred strategy, leading to better management of COPD [3, 12, 13].

Previous research by Vanfleteren *et al.* [5] explored comorbidity profiling in COPD using self-organising maps (SOMs) to identify clusters that might relate to different pathophysiological pathways. However, translating these findings into clinical practice requires reproducibility. Although unsupervised clustering is commonly used for heterogeneity assessment in COPD research [14–18], systematic reviews have found inconsistencies in the results. These inconsistencies often stem from cohort selection biases and varied methodologies across studies, raising concerns about the reliability of the identified comorbidity profiles [19–21].

To address these gaps and extend on the foundational research, this study applied uniform clustering analyses to two large prospective cohorts of COPD patients. The aim was to identify comorbidity profiles and compare results across cohorts in terms of disease severity, clinical characteristics and outcomes, to reveal clinically meaningful phenotypes.

## Material and methods

### Study cohorts

The analyses were based on data from two observational COPD cohorts: the Evaluation of COPD Longitudinally to Identify Predictive Surrogate End-points study (ECLIPSE; NCT00292552) and the Groningen Severe COPD Cohort (GSCC; NCT04023409).

The study design of ECLIPSE, which enrolled 2164 COPD patients, has been published previously [22]. In brief, ECLIPSE is a multicentre study with recruitment from 2005 to 2010. Participants were evaluated at 3, 6 and every subsequent 6 months for 3 years after enrolment.

Data for GSCC were collected at enrolment from 1030 severe COPD patients referred to the University Medical Center Groningen for bronchoscopic lung volume reduction (LVR) consultation between 2014 and 2019. The inclusion criteria required a post-bronchodilator forced expiratory volume in 1 s (FEV_1_) to forced vital capacity (FVC) ratio of ≤0.7.

Written informed consent was obtained from all participants prior to participation, with approval from Ethics Committees of all involved institutions.

To ensure comparability between the cohorts, the GSCC underwent additional screening to align with ECLIPSE inclusion criteria [22]: I) patients aged 40–75 years; II) post-bronchodilator FEV_1_ <80% of the reference value; and III) current or former smokers with a history ≥10 pack-years. Key exclusion criteria for this additional screening included severe α_1_-antitrypsin deficiency, significant inflammatory disease history and a recent diagnosis of cancer.

### Measurements

At enrolment, patients’ demographics and smoking status were recorded, and the following assessments were performed: lung function measurements (post-bronchodilator spirometry); questionnaires (modified Medical Research Council (mMRC) and the COPD-version of the St. George's Respiratory Questionnaire (SGRQ-C)); and nutritional status (body mass index (BMI) and fat-free mass index (FFMI)). Acute exacerbations of COPD (AECOPD) requiring medication and/or hospitalisation in the year prior to study entry were recorded. All patients underwent a computed tomography (CT) scan of the chest, and the extent of emphysema was quantified using density mask analysis with a threshold of −950 Hounsfield units (HU) [23]. The GOLD ABE assessment score was determined according to 2023 guidelines [6], and spirometry reference values were calculated using the Global Lung Initiative (GLI) 2012 equation [24]. Participants in both cohorts were followed for survival data for a minimum of 3 years.

### Definitions of comorbidities

13 comorbidities were selected for analysis: 10 from the American Thoracic Society-Division of Lung Diseases (ATS-DLD-78) questionnaire and three based on nutritional status. Each comorbidity needed to be present in >5% of ECLIPSE participants, and comorbidity data had to be available in both cohorts. The comorbidities included were: obesity, underweight, muscle wasting, heart failure, heart attack, diabetes, peptic ulcer, osteoporosis, osteoarthritis, depression, anxiety, hypertension and history of asthma. In ECLIPSE, comorbidities were identified using self-reported data from the ATS-DLD-78 questionnaire or through nutritional status cut-offs. For the GSCC, comorbidity data came from self-reports, nutritional cut-offs or a review of medical histories (see online supplementary material, Section 1 for details).

Patients missing data on >1 of the 13 comorbidities were excluded from the study. One-hot encoded tables were generated, encoding the comorbidity data as missing, 1 or 0. No imputation of comorbidity data was performed. Patients with none of the selected comorbidities were withheld from the clustering and served as a reference group.

### Unsupervised machine learning

Using the Kohonen package [25] (R version 4.2.2), SOMs were generated to create an ordered representation based on the one-hot encoded comorbidity tables. Hereafter, clusters were superimposed onto the distance arrays of the SOM nodes using the Ward Cluster algorithm [26]. For details on SOMs configuration, determining cluster assignment and stability testing, refer to the online supplementary material, Section 2. Once the optimal cluster assignment was determined, heatmaps were used to visualise the separation of clusters. To explore the possibility of patients belonging to multiple clusters, additional analysis was conducted (online supplementary material, section 2.4).

### Statistical analysis

Summary variables for comorbidities and clinical characteristics are presented for each cohort and cluster. Continuous variables are shown as mean±standard deviation, while categorical variables are listed as total numbers and frequencies. Comparisons of continuous variables between clusters were conducted using one-way analysis of variance (ANOVA), whereas a two-sample unpaired t-test was used for comparing the cohorts. Categorical variables were analysed with the chi-squared test. Survival analyses were performed using Kaplan–Meier curves and Cox proportional hazards (PH) models (see online supplementary material, section 3 for details on testing the PH assumption). Results are presented in both univariate- and covariate-adjusted forms, with adjustments for age, sex, BMI, FEV_1_ % predicted and emphysema extent. To reduce loss-to-follow-up, mortality data were capped at 3 years (1095 days) aligning with the original follow-up time of ECLIPSE. A time-to-first-severe-exacerbation analysis was performed in ECLIPSE (online supplementary material, section 4). p-values <0.05 were considered significant. No adjustments for multiple comparisons were performed. All statistical analyses were conducted with R version 4.2.2.

## Results

### Patient characteristics

**FIGURE 1:**
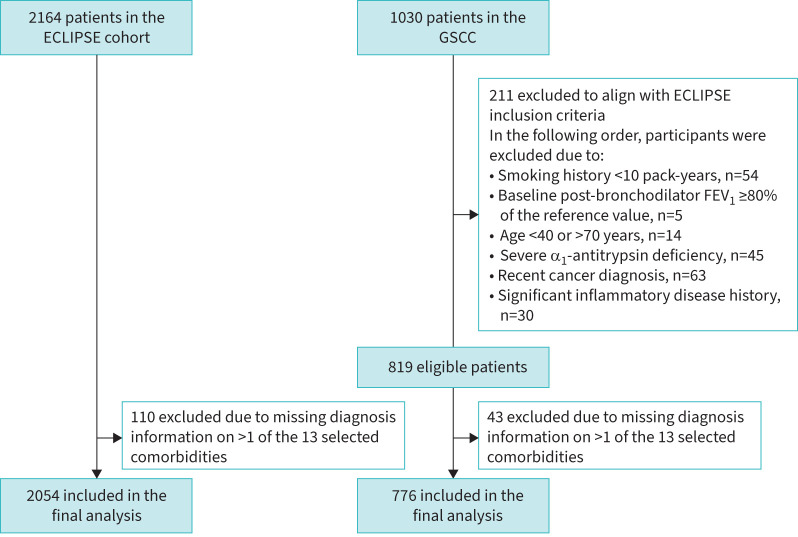
A flowchart for selection of patients from the ECLIPSE cohort and the Groningen Severe COPD Cohort (GSCC). FEV_1_: forced expiratory volume in 1 s.

The final study population consisted of 2054 and 776 COPD patients from ECLIPSE and GSCC, respectively (figure 1). Table 1 presents the baseline characteristics, indicating significant differences between the two cohorts. The GSCC displayed more advanced disease features, with lower FEV_1_ % and FEV_1_/FVC ratios, and higher rates of AECOPD, SGRQ-C total scores, mMRC scores and emphysema extent. Additionally, a larger proportion of GSCC patients were classified in GOLD stage E, the most severe category of COPD. The GSCC also showed a higher percentage of female patients, a lower proportion of current smokers and a slightly lower nutritional status (BMI and FFMI) compared to the ECLIPSE cohort.

**TABLE 1:** Baseline patient characteristics of the two cohorts

Clinical characteristics	ECLIPSE	GSCC	p-value
**Patients n**	2054	776	
**Age years**	63.3±7.1	61.4±6.9	<0.001
**Male sex**	1337 (65.1)	266 (34.3)	<0.001
**BMI kg·m^−2^**	26.5±5.7	24.4±4.3	<0.001
**FFMI kg·m^−2^**	17.7±3.4	15.6±2.0	<0.001
**Current smoker**	746 (36.3)	15 (1.9)	<0.001
**Pack-years**	48.7±27.1	41.4±18.1	<0.001
**FEV_1_ % predicted**	43.7±14.9	29.6±10.0	<0.001
**FEV_1_/FVC ratio**	0.45±0.1	0.31±0.07	<0.001
**mMRC score**	1.66±1.1	2.63±0.63	<0.001
**SGRQ-C total score**	49.7±20.4	58.9±13.2	<0.001
**GOLD stage**			
** **A	805 (40.0)	2 (0.3)	<0.001
** **B	765 (38.0)	301 (40.8)	
** **E	443 (22.0)	434 (58.9)	
**AECOPD** ^#^	0.78±0.98	2.3±2.1	<0.001
**Emphysema (−950** **HU)**	17.7±12.3	36.4±8.24	<0.001

Summary statistics are expressed as mean±sd for continuous variables, while categorical variables are presented as n (%). Differences between groups were assessed using the two-sample unpaired t-test for continuous variables and the chi-squared test for categorical variables. ECLIPSE: the Evaluation of COPD Longitudinally to Identify Predictive Surrogate End-points study; GSCC: Groningen Severe COPD Cohort; BMI: body mass index; FFMI: fat-free mass index; FEV_1_: forced expiratory volume in 1 s; FVC: forced vital capacity; mMRC: Modified Medical Research Council; SGRQ-C: COPD-specific version of the St George's Respiratory Questionnaire; GOLD: Global Initiative for Chronic Obstructive Lung Disease; AECOPD: acute exacerbations of COPD; HU: Hounsfield units. ^#^: mean±sd number of moderate/severe exacerbations of COPD recorded in the year prior to study entry.

### Prevalence of comorbidities

**FIGURE 2:**
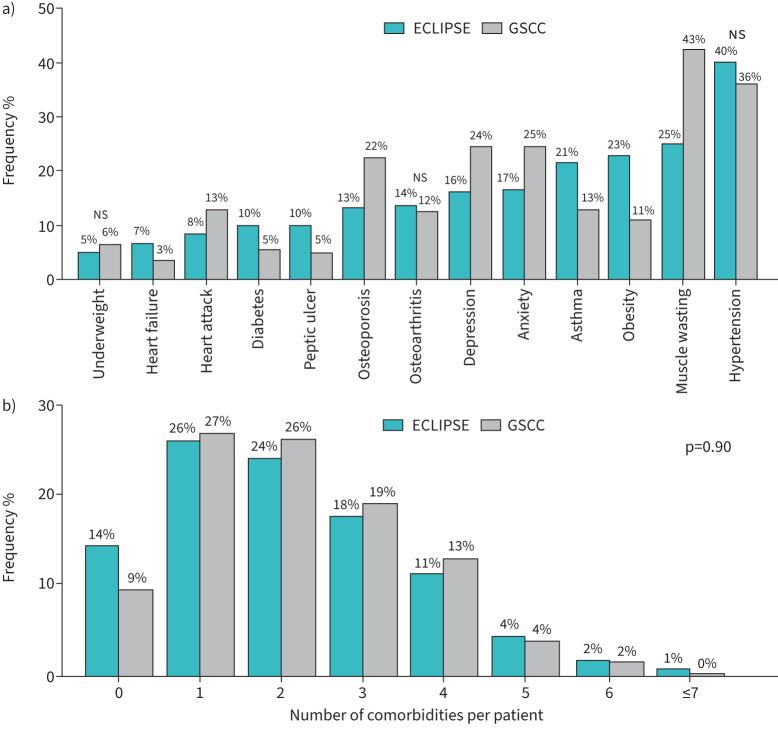
a) Frequency of comorbidities observed in 2054 COPD patients from ECLIPSE and 776 patients from Groningen Severe COPD Cohort (GSCC). Significant differences were observed in the presence of all comorbidities between the cohorts, as determined by the chi-squared test, except for those marked as not significant (NS). b) Frequency of the total number of comorbidities per patient. No significant difference in the frequency between the cohorts was observed, as determined by the chi-squared test.

Figure 2a shows the prevalence of comorbidities in each cohort, with frequencies ranging from 3% to 43%. Hypertension and muscle wasting were the most common comorbidities in both cohorts. Heart failure, diabetes, peptic ulcer, history of asthma and obesity were significantly more prevalent in the ECLIPSE cohort, while heart attack, osteoporosis, depression, anxiety and muscle wasting were significantly more prevalent in the GSCC. In both cohorts, most patients had at least one comorbidity: 86% in ECLIPSE and 91% in GSCC (figure 2b). Over half of the patients experienced at least two comorbidities alongside COPD. 296 (14%) patients in ECLIPSE and 71 (9%) patients in GSCC had none of the 13 selected comorbidities.

### Clusters of comorbidities

**FIGURE 3:**
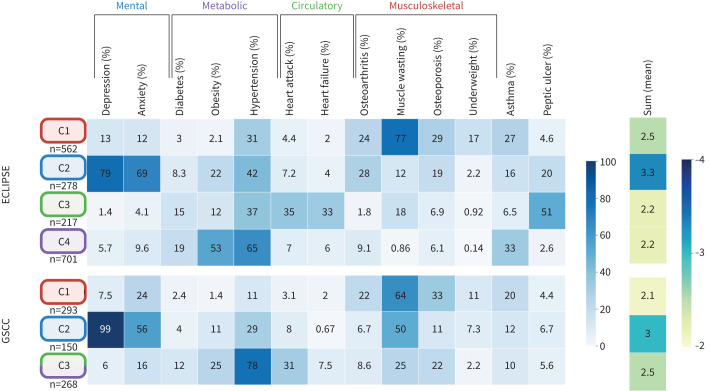
The frequencies of comorbidities identified in each cluster from ECLIPSE and Groningen Severe COPD Cohort (GSCC). Each row represents a cluster, and the columns indicate the prevalence frequency (%) of the comorbidities within that respective cluster. The rightmost column provides the mean count of comorbidities per patient in each cluster.

The SOM-Ward clustering analysis revealed comorbidity profiles within ECLIPSE and GSCC, with four clusters identified in ECLIPSE and three in GSCC (figure 3; online supplementary material, Section 5). Two clusters (C1 and C2) showed high similarity across both cohorts, while the third cluster in GSCC appeared to combine features of the third (C3) and fourth (C4) clusters from ECLIPSE.

C1 (Musculoskeletal) had a higher prevalence of underweight, muscle wasting and osteoporosis, but lower incidences of obesity, diabetes and hypertension. C2 (Mental) had the highest proportion of patients with psychological disorders, such as anxiety and depression, and the highest overall count of comorbidities. In ECLIPSE, C3 (Circulatory) was characterised by a higher prevalence of heart attack and heart failure, whereas C4 (Metabolic) included more patients with obesity, diabetes and hypertension, but fewer with underweight and muscle wasting. The third cluster (C3: Circulatory/Metabolic) in GSCC reflected elements of both the circulatory and metabolic cluster profiles identified in ECLIPSE.

### Patient characteristics of each comorbidity cluster

Patients’ demographics varied significantly among clusters, with comparable trends observed across cohorts (tables 2 and 3). The musculoskeletal cluster primarily consisted of females with lower BMI and FFMI. The mental cluster also had a higher proportion of females and was, on average, younger. In contrast, the reference group (no comorbidities), and the circulatory and metabolic cluster (combined into one cluster in GSCC), consisted of predominantly older males with above-average FFMI.

**TABLE 2:** Clinical characteristics of the patients in the four clusters identified in ECLIPSE

	Reference: No comorbidities	C1: Musculoskeletal	C2: Mental	C3: Circulatory	C4: Metabolic	p-value
**Patients, n**	296	562	278	217	701	
**Age years**	62.6±7.3	63.5±7.1	61.7±7.0^#^	64.5±6.7 ^¶^	63.9±7.0 ^¶^	<0.001
**Male sex**	246 (83.1)^¶^	240 (42.7)^#^	151 (54.3)^#^	179 (82.5)^¶^	521 (74.3)^¶^	<0.001
**BMI kg·m^−2^**	25.3±2.5^#^	22.3±4.2^#^	26.7±5.0	26.1±4.4	30.5±5.6^¶^	<0.001
**FFMI kg·m^−2^**	18.3±1.9^¶^	14.4±2.6^#^	17.8±2.9	18.3±3.0^¶^	19.9±2.9^¶^	<0.001
**Current smoker**	118 (39.9)	238 (42.3)^¶^	114 (41.0)	76 (35.0)	200 (28.5)^#^	<0.001
**Pack-years**	48.8±26.4	45.2±23.7^#^	54.9±28.9^¶^	46.8±28.0	49.5±28.6	<0.001
**FEV_1_ % predicted**	45.3±14.5^¶^	40.6±14.5^#^	42.3±14.9	45.2±15.8	45.5±14.5^¶^	<0.001
**FEV_1_/FVC ratio**	0.4±0.1	0.4±0.1^#^	0.4±0.1	0.5±0.1^¶^	0.5±0.1^¶^	<0.001
**mMRC score**	1.4±1.0^#^	1.7±1.1	1.9±1.1^¶^	1.7±1.0	1.7±1.0	<0.001
**SGRQ-C score**						
** **Activity	54.4±25.2^#^	66.0±24.4^¶^	72.6±22.5^¶^	61.3±25.2	62.5±25.6	<0.001
** **Impact	32.2±22.2^#^	37.7±21.6	44.9±20.8^¶^	38.0±21.0	38.3±22.4	<0.001
** **Symptoms	58.1±21.9	59.8±21.2	64.2±21.3^¶^	59.5±21.5	59.8±22.3	0.012
** **Total	43.5±20.7^#^	50.2±19.6	56.7±18.5^¶^	48.9±19.9	49.3±20.8	<0.001
**GOLD stage**						
** **A	149 (51.2)^¶^	213 (38.7)	90 (33.1)^#^	83 (38.6)	270 (39.5)	0.001
** **B	90 (30.9)^#^	205 (37.2)	107 (39.3)	88 (40.9)	275 (40.2)	
** **E	52 (17.9)^#^	113 (24.1)	75 (27.6)^¶^	44 (20.5)	139 (20.3)	
**AECOPD^+^**	0.6±0.9 ^#^	0.9±1.0^¶^	0.9±1.1^¶^	0.8±1.0	0.7±1.0^#^	0.002
**Emphysema (−950** **HU)**	16.7±12.1	21.6±13.8^¶^	19.1±12.5	16.4±11.5	14.7±10.1^#^	<0.001

Summary statistics are expressed as mean±sd for continuous variables, while categorical variables are presented as n (%). Differences between clusters were assessed using one-way analysis of variance for continuous variables and the chi-squared test for categorical variables. ECLIPSE: the Evaluation of COPD Longitudinally to Identify Predictive Surrogate End-points study; BMI: body mass index; FFMI: fat-free mass index; FEV_1_: forced expiratory volume in 1 s; FVC: forced vital capacity; mMRC: Modified Medical Research Council; SGRQ-C: COPD-specific version of the St. George's Respiratory Questionnaire; GOLD: Global Initiative for Chronic Obstructive Lung Disease; AECOPD: acute exacerbations of COPD; HU: Hounsfield units. ^#^: significantly less prevalent compared with the rest of the cohort population; ^¶^: significantly more prevalent compared with the rest of the cohort population; **^+^**: mean±sd number of moderate/severe exacerbations of COPD recorded in the year prior to study entry.

**TABLE 3:** Clinical characteristics of the patients in the three clusters identified in GSCC

	Reference: No comorbidities	C1: Musculoskeletal	C2: Mental	C3: Circulatory/Metabolic	p-value
**Patients, n**	71	293	150	268	
**Age years**	62.3±6.4	60.7±7.1	59.2±7.1^#^	63.2±6.7^¶^	<0.001
**Male sex**	40 (56.3)^¶^	81 (27.6)^#^	34 (22.7)^#^	115 (42.9)^¶^	<0.001
**BMI kg·m^−2^**	24.8±2.2	22.6±3.5^#^	24.2±4.3	26.3±4.5^¶^	<0.001
**FFMI kg·m^−2^**	16.7±1.0^¶^	14.8±1.5^#^	15.2±1.9^#^	16.6±2.2^¶^	<0.001
**Current smoker**	1 (1.4)	7 (2.4)	4 (2.7)	3 (1.1)	0.615
**Pack-years**	41.7±14.5	39.3±15.9^#^	41.2±18.8	43.7±20.5^¶^	0.036
**FEV_1_ % predicted**	29.4±9.7	29.2±9.3	29.5±11.7	30.2±9.9	0.677
**FEV_1_/FVC ratio**	0.3±0.1	0.3±0.1	0.3±0.1	0.3±0.1	0.287
**mMRC score**	2.4±0.6^#^	2.6±0.6	2.7±0.6	2.6±0.6	0.022
**SGRQ-C score**					
** **Activity	81.7±13.1	83.7±11.8	84.0±13.6	83.1±11.5	0.568
** **Impact	42.8±14.7^#^	48.0±16.7	50.4±16.6^¶^	45.0±17.9^#^	0.003
** **Symptoms	48.6±20.2	53.9±19.0	53.6±20.0	52.1±19.7	0.194
Total	55.6±12.7^#^	59.8±13.0	61.1±12.9^¶^	57.7±13.7	0.009
**GOLD stage**					
** **A	0 (0)	1 (0.4)	0 (0)	1 (0.4)	0.607
** **B	30 (46.2)	102 (36.6)	62 (43.4)	110 (43.1)	
** **E	35 (53.8)	176 (63.1)	81 (56.6)	144 (56.5)	
**AECOPD^+^**	2.0±1.8	2.4±2.0	2.5±2.5	2.2±2.0	0.377
**Emphysema (−950** **HU)**	35.8±8.6	38.1±7.9^¶^	36.1±8.1	34.8±8.2^#^	<0.001

Summary statistics are expressed as mean±sd for continuous variables, while categorical variables are presented as n (%). Differences between clusters were assessed using one-way analysis of variance for continuous variables and the chi-squared test for categorical variables. GSCC: Groningen Severe COPD Cohort; BMI: body mass index; FFMI: fat-free mass index; FEV_1_: forced expiratory volume in 1 s; FVC: forced vital capacity; mMRC: Modified Medical Research Council; SGRQ-C: COPD-specific version of the St. George's Respiratory Questionnaire; GOLD: Global Initiative for Chronic Obstructive Lung Disease; AECOPD: acute exacerbations of COPD; HU: Hounsfield units. ^#^: significantly less prevalent compared with the rest of the cohort population; ^¶^: significantly more prevalent compared with the rest of the cohort population; ^+^: mean±sd number of moderate/severe exacerbations of COPD recorded in the year prior to study entry.

In ECLIPSE, distinct differences in disease severity were evident among the clusters (table 2). The reference group, which had no comorbidities, displayed high (*i.e.*, favourable) spirometry measures and low scores on the SGRQs-C and mMRC questionnaires, reflecting better overall health and less impaired breathing. The reference group also experienced fewer AECOPD and had fewer patients in the GOLD stage E category, suggesting less advanced disease. Conversely, the musculoskeletal and mental cluster showed more advanced disease features. The musculoskeletal cluster had lower spirometry measures, while the mental cluster had higher scores in mMRC and all SGRQ-C dimensions, along with significantly higher AECOPD rate in both. The circulatory and metabolic cluster had relatively preserved spirometry measures, with the metabolic cluster additionally having a significantly lower AECOPD rate and less emphysema.

In GSCC, there were minimal variations in disease severity across the comorbidity clusters (table 3). The mental cluster showed higher SGRQ-C impact and total scores, while the reference group had overall lower SGRQ-C scores. Emphysema extent was higher in the musculoskeletal cluster and lower in the combined circulatory/metabolic cluster.

### Mortality analyses

**FIGURE 4:**
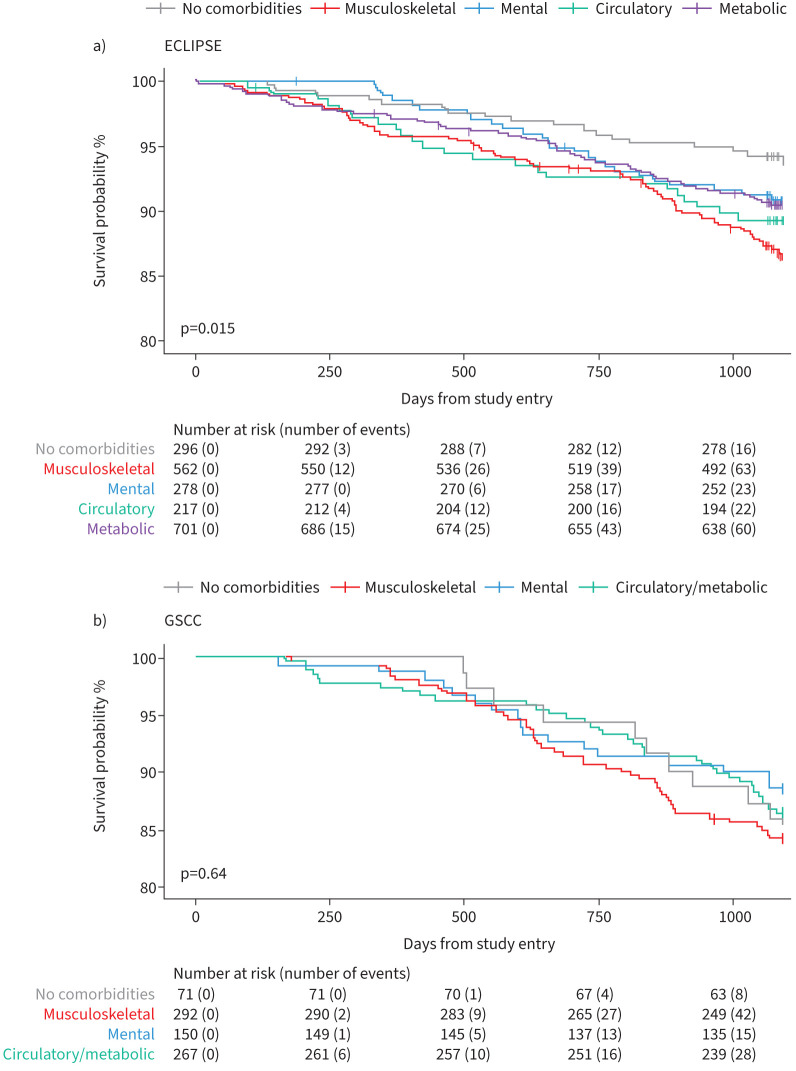
a) Kaplan–Meier curves showing survival over 1095 days (3 years) in the reference group without comorbidities and the four clusters across the ECLIPSE. b) Kaplan–Meier curves showing survival over 1095 days (3 years) in the reference group without comorbidities and the three clusters across the Groningen Severe COPD Cohort (GSCC).

Mortality analyses showed different outcomes between the two cohorts. The ECLIPSE cohort had a 90% survival rate after 1095 days following study entry, while GSCC had an 86% survival rate (online supplementary material, section 6). In ECLIPSE, significant differences in unadjusted mortality risk were found between clusters (log-rank p=0.015; figure 4a). The musculoskeletal cluster was associated with significantly higher mortality risk (hazard ratio (HR) 2.27; 95% CI 1.36–3.80; p=0.002; table 4) compared to patients without comorbidities. The circulatory and metabolic clusters also showed trends toward increased mortality risk. After covariate adjustments, the HR for the musculoskeletal cluster remained significantly elevated (HR 2.30; 95% CI 1.26–4.13; p=0.006), while the increased mortality trends for the circulatory and metabolic clusters diminished. In contrast, GSCC showed no significant differences in mortality risk among the comorbidity clusters (figure 4b and table 4).

**TABLE 4:** Mortality analyses

	ECLIPSE				GSCC		
	C1: Musculoskeletal	C2: Mental	C3: Circulatory	C4: Metabolic	C1: Musculoskeletal	C2: Mental	C3: Circulatory/Metabolic
**Univariate**	2.27 (1.36–3.80) p=0.002	1.50 (0.82–2.75) p=0.19	1.80 (0.97–3.33) p=0.06	1.58 (0.94–2.65) p=0.09	1.14 (0.58–2.28) p=0.69	0.81 (0.37–1.77) p=0.60	0.96 (0.48–1.94) p=0.91
**Multivariate**	2.30 (1.26–4.13) p=0.006	1.36 (0.68–2.70) p=0.38	1.67 (0.84–3.33) p=0.14	1.39 (0.76–2.52) p=0.28	1.10 (0.52–2.31) p=0.80	0.88 (0.38–2.06) p=0.77	1.07 (0.51–2.25) p=0.85

Patients with no comorbidities were used as reference. Data are presented as hazard ratios with 95% confidence intervals and p-values. Multivariate mortality analyses are adjusted for age, sex, body mass index, % predicted forced expiratory volume in 1 s and emphysema extent (−950 Hounsfield units). ECLIPSE: Evaluation of COPD Longitudinally to Identify Predictive Surrogate End-points study; GSCC: Groningen Severe COPD Cohort.

## Discussion

In two real-world cohorts of COPD patients with varying disease severity, comparable clusters were identified. These clusters differed significantly in patient characteristics and symptom burden. To our knowledge, this is the first study to use identical methodologies across multiple COPD cohorts, identifying comorbidity patterns throughout all stages of the disease (GOLD stage A, B and E) and validating the findings in a severe COPD subpopulation (GOLD stage E-dominant). This approach highlights the generalisability of the identified comorbidity clusters and addresses the concerns about cohort selection bias, reproducibility and methodological variability.

Community-wide acceptance of comorbidity profiles could pave the way to identify affected pathways, where genes and proteins interact to form distinct disease modules [27]. This may uncover clinically significant endotypes, which can define more “typical” patients rather than extremes, and aid in predicting which individuals are more likely to respond to specific drugs or interventions. Future research should link various “omics” fields with comorbidity cluster analyses to better understand the potential pathways involved.

In this study, a musculoskeletal cluster was identified in both cohorts, characterised by worsened spirometry measures and increased emphysema. This cluster resembles the historically described “pink puffer” phenotype [28]. The mechanisms driving sarcopenia remain largely unknown. One hypothesis is that local pro-inflammatory cytokines and chemokines enter the bloodstream, initiating and sustaining systemic inflammation [29]. Additionally, breakdown products from the extracellular matrix (ECM), including fibronectin, decorin and biglycan [30, 31], may also enter the circulation and contribute to COPD comorbidities. Future research should investigate whether systemic cytokine profiles and ECM breakdown products are associated with this cluster. Some associations were observed between the musculoskeletal cluster and higher mortality, which highlights the need for early intervention before sarcopenia becomes fully established. Identifying patients at risk, potentially through prognostic biomarkers, could help assess whether timely treatments improve survival, though this could not be explored with the available cohorts.

The mental cluster identified in both cohorts, characterised by a higher prevalence of anxiety and depression, represents a less recognised COPD phenotype. Although there is growing attention to mental health in COPD patients [32, 33], this phenotype remains largely underexplored. Patients in this cluster did not show significant differences in objective measures like spirometry but reported poorer subjective assessments and had a higher AECOPD rate. This highlights the need for early screening for depression and anxiety in COPD patients to identify this specific phenotype (“frequent exacerbators”) and facilitate timely intervention.

In the ECLIPSE cohort, both a metabolic and a circulatory cluster were identified, categorising predominantly older males with less severe pulmonary impairment into two phenotypes: the well-known “blue bloater” [28] and a less characterised cardiopulmonary subgroup. This separation is vital for managing COPD comorbidities and tailoring medical therapies. It differentiates between cardiovascular issues potentially related to obesity and those stemming from a shared disease pathway or the systemic effects of pro-inflammatory mediators. Therefore, one group might need anti-inflammatory drugs alongside standard care, while the other may benefit more from weight management interventions and might not respond as well to anti-inflammatory medications. In the GSCC, the metabolic and circulatory clusters were combined, likely due to a low number of patients with obesity.

Previous analyses of COPD clustering have used heterogeneous lists of chronic diseases [5, 16–18]. While these studies have consistently identified clusters, the profiles and results differ. However, some of the findings in this paper echo previous research.

Vanfleteren *et al.* [5] identified five clusters in their study of 255 COPD patients using the SOM-Ward method: less comorbidity, cardiovascular, cachectic, metabolic and psychological. This study's clusters are similar, though the “less comorbidity” cluster was not identified, likely due to the exclusion of patients without comorbidities. Vanfleteren *et al.* found similar disease severity across clusters, possibly due to selection bias as the cohort included only stable-state COPD patients undergoing pulmonary rehabilitation, which limits generalisability. Furthermore, no outcome data were available, limiting the evaluation of the comorbidities cluster on disease progression.

Vikjord *et al.* [17] employed similar methodology in a study of 2076 COPD-suspected patients from a Norwegian longitudinal survey and identified comparable clusters. Vikjord *et al*.'s study faced limitations, including potential overdiagnosis of COPD-suspected patients and a healthy-selection bias, as indicated by unusually high spirometry measures [34, 35], which limits its applicability to diagnosed COPD populations. However, unlike Vanfleteren *et al*., Vikjord *et al*. had outcome data available, linking the psychological and cachectic clusters to a significantly higher all-cause mortality risk. These findings were only modestly replicated in this study, with the musculoskeletal cluster (cf. the cachectic cluster) being associated with increased mortality (HR 1.26–4.13) solely in the ECLIPSE cohort, which contrasts with literature linking both sarcopenia and mental disorders to higher COPD mortality [36, 37]. This discrepancy may result from differences in follow-up time, as the presented study had a shorter follow-up compared to others, a discrepancy also shown in the literature [4].

### Methodological limitations

Several limitations were considered. First, the ECLIPSE study lacked comprehensive objective measures for comorbidities, relying primarily on binary questionnaires. This limited the assessment to the 13 comorbidities recorded at study enrolment, excluding prevalent diseases such as obstructive sleep apnoea, atherosclerosis and chronic kidney disease.

Another limitation is the absence of data on the duration and severity of comorbidities, which could lead to misclassification. Patients with comorbidities diagnosed early in life and well managed might be grouped with those newly diagnosed with more severe conditions, affecting long-term outcomes. Additionally, the lack of follow-up data on emerging comorbidities prevented assessment of the stability of the clusters over time.

The hierarchical clustering approach used in this study enforces mutually exclusive cluster assignments, which may misclassify patients with comorbidities spanning multiple categories. In such cases, the algorithm assigns patients to a single cluster based on the “strongest” comorbidity, which is model- and cohort-dependent. This may introduce variability in patient classification. Fuzzy clustering [38] could address this by allowing overlapping memberships, though final assignments would still involve subjectivity.

Despite the GSCC having more advanced disease at study entry, only a modest difference in mortality rates was observed between the two cohorts. This could be due to the relatively short follow-up period, which was chosen to minimise patient loss-to-follow-up. The lack of mortality differences between clusters in the GSCC may also relate to an uneven LVR treatment distribution, though this hypothesis could not be tested due to missing information.

Additional outcome data beyond all-cause mortality would be valuable, as COPD management encompasses more than just survival. Intervention studies, like those by Mesquita *et al*. [39], examining the impact of comorbidity clusters in pulmonary rehabilitation, are crucial in understanding how these profiles affect patient outcomes. Expanding research to treatments like biologics and ventilation may help promote the use of comorbidity profiles in personalised disease management.

In conclusion, this study confirms comorbidity clusters linked to different patient subgroups. These findings improve our understanding of the comorbidity landscape in COPD and could potentially inform future treatment guidelines. Further research, including biomarker profiling and intervention studies, could provide deeper insights into the underlying mechanisms and better treatment options for these comorbidity clusters.

## Data Availability

Anonymised individual participant data related to the ECLIPSE dataset are available to researchers upon approval of a data access request *via* dbGaP. Data from the GSCC that support the findings of this study are available from Jorine E. Hartman (j.hartman@umcg.nl) upon reasonable request. All analyses were conducted using publicly available software, with relevant parameters detailed in the methods section or in the supplementary material. The code used for this study is specifically tailored to the GSCC and ECLIPSE data, and cannot function independently without access to these datasets. The corresponding author welcomes being contacted for additional information to help reproduce the results presented in this paper.

## Supplementary material

**Please note:** supplementary material is not edited by the Editorial Office, and is uploaded as it has been supplied by the author. Supplementary material 01289-2024.SUPPLEMENT
