# Supplementary material for: Comorbidity profiles in chronic obstructive pulmonary disease: a multicohort study
Source: ERJ Open Res. 2025 Oct 27;11(5):01289-2024. doi: 10.1183/23120541.01289-2024 (PMC12557400; doi:10.1183/23120541.01289-2024)
Supplement: Supplementary file 1 [file 01289-2024.SUPPLEMENT.pdf]

SUPPLEMENTARY MATERIAL

Supplementary material for the article:

"Comorbidity Profiles in Chronic Obstructive Pulmonary Disease: a multicohort study"

by L Egerod, EAMD ter Haar, MA Karsdal, DJ Leeming, CB Nanthakumar, JC Yates, DJ Slebos, SD Pouwels, JE Hartman, and JMB Sand.

This appendix consists of the following sections:

Section 1: Definition of Comorbidities *page 2*

Section 2: Configuration of the Self-organizing Maps *page 4*

Section 3: Testing the Proportional Hazards Assumption *page 20*

Section 4: Time-to-first-severe-exacerbation *page 25*

Section 5: Comorbidities Identified in Each Cluster *page 27*

Section 6: Kaplan-Meier curve comparing ECLIPSE and GSCC *page 29*

References *page 30*

Section 1: Definition of comorbidities in the Evaluation of COPD Longitudinally to Identify Predictive Surrogate Endpoints (ECLIPSE) cohort and the Groningen Severe COPD Cohort (GSCC)

Suppl. Table S1: In the ECLIPSE cohort, comorbidities were obtained from either the self-reported questionnaire (ATS-DLD-78) or based on predefined cut-offs related to nutritional status. For the GSCC, comorbidities assessment was based on self-reported questionnaires, the predefined nutritional status cut-offs, and a review of the patients' medical history

Cohort	Comorbidity	Defined by
ECLIPSE	Anxiety	ATS-DLD-78 Questionnaire: "Ever been told had anxiety or panic attacks"
	Asthma	ATS-DLD-78 Questionnaire: "Asthma confirmed by a doctor"
	Depression	ATS-DLD-78 Questionnaire: "Ever been told had depression requiring treatment"
	Diabetes	ATS-DLD-78 Questionnaire: "Ever been told had diabetes"
	Heart failure	ATS-DLD-78 Questionnaire: "Ever been told had heart failure"
	Heart attack	ATS-DLD-78 Questionnaire: "Ever been told had heart attack"
	Hypertension	ATS-DLD-78 Questionnaire: "Ever been told had hypertension"
	Muscle wasting	Cutoff criteria: FFMI < 16 [Males], FFMI < 15 [Females]
	Obesity	Cutoff criteria: BMI >= 30
	Osteoarthritis	ATS-DLD-78 Questionnaire: "Ever been told had osteoarthritis"
	Osteoporosis	ATS-DLD-78 Questionnaire: "Ever been told had osteoporosis"
	Peptic ulcer	ATS-DLD-78 Questionnaire: "Ever been told had peptic ulcer"
	Underweight	Cutoff criteria: BMI < 18.5
GSCC	Anxiety	Reported in their medical history or reported by the patient in a self-administered questionnaire.<br>Question: "Can you check (tick the box) which of the following conditions you have or have had? Answer: Social anxiety disorder, agoraphobia, panic disorder, obsessive-compulsive disorder, or any other anxiety disorder"
	Asthma	Reported by the patient in a self-administered questionnaire.<br>Question: "Have you ever had asthma? Answer: Yes"
	Depression	Reported in their medical history or reported by the patient in a self-administered questionnaire.<br>Question: "Can you check (tick the box) which of the following conditions you have or have had? Answer: Depression"
	Diabetes	Reported in their medical history or reported by the patient in a self-administered questionnaire.<br>Question: "Do you have diabetes? Answer: Yes"

	Heart failure	Left ventricular ejection fraction (LVEF) <55% with concomitant use of heart failure medication OR when reported by the patient in the self-administered questionnaire.<br>Question: “Do you have heart failure (reduced heart pumping function)? Answer: Yes”
	Heart attack	Reported in their medical history or reported by the patient in a self-administered questionnaire.<br>Question: “Have you ever had an angioplasty (widening of a blood vessel with a balloon) and/or bypass surgery? Answer: Yes” OR Question: “Have you ever had a heart attack? Answer: Yes”
	Hypertension	Defined as a systolic blood pressure above 140mmHg, or a diastolic blood pressure above 90mmHg, AND the concomitant use of antihypertensive drugs OR when reported by the patient in the self-administered questionnaire.<br>Question: “How are you being treated for high blood pressure? Answer: with medication / with both (low sodium-diet and medication) OR Question: “Have you ever had a high blood pressure?” Answer: Yes.
	Muscle wasting	Cutoff criteria: FFMI < 16 [Males], FFMI < 15 [Females]
	Obesity	Cutoff criteria: BMI >= 30
	Osteoarthritis	Reported by the patient in a self-administered questionnaire.<br>Question: “Can you check (tick the box) which of the following conditions you have or have had? Answer: Arthrosis/osteoarthritis”
	Osteoporosis	Reported in their medical history or reported by the patient in a self-administered questionnaire.<br>Question: “Can you check (tick the box) which of the following conditions you have or have had? Answer: Osteoporosis”
	Peptic ulcer	Reported by the patient in a self-administered questionnaire.<br>Question: “Can you check (tick the box) which of the following conditions you have or have had? Answer: Stomach ulcer”
	Underweight	Cutoff criteria: BMI < 18.5

BMI = Body mass index; FFMI = Fat free mass index

Section 2: Configuration of the self-organizing maps, the process for determining optimal cluster assignment, and additional stability testing of the clusters

Self-organizing maps (SOMs) were implemented by R v.4.2.2 using the publicly available R library: *kohonen*[1] (v.3.0.11). The *supersom*-extension was utilized to handle missing values without imputation. Clusters were superimposed onto the SOMs using the *Ward.D* (Ward Cluster) algorithm.[2] Default hyperparameters were applied, with modifications to grid size (*grid*), iteration length (*rlen*), and the number of clusters (*k_cluster*).

The *grid* was calculated using the heuristic formula: $5 \times n^{0.54321}$, where n represents the number of records in the dataset.[3] The *rlen* and *k_cluster* parameters were optimized for each cohort, as detailed in *sections 2.1.1* and *2.1.2*.

2.1 SOMs for the cohorts

Optimized parameters used in the ECLIPSE SOM: *grid=(16,13), rlen=500, k_cluster=4.*

Optimized parameters used in the GSCC SOM: *grid=(13,10), rlen=700, k_cluster=3.*

2.1.1 Iteration length (*rlen*) optimization

During SOM training, the distance between each node's weights and the samples it represents decreases progressively with each iteration over the full dataset. This distance should stabilize at a minimum plateau, beyond which further iterations yield negligible improvements.[4] Monitoring this progress over time through a plot can indicate whether convergence has been achieved. If the curve continues to decrease, additional iterations are necessary.

ECLIPSE: See ***Suppl. Figure S1A*** for monitoring of the progress for the ECLIPSE cohort. The plots demonstrate that the default value of 100 iterations in the *kohonen* package was insufficient, as the curve was still decreasing. At *rlen*=200, a small plateau began to appear, but a slight decrease within the plateau was still observed at *rlen*=300 and *rlen*=400. By *rlen*=500, this decrease had stabilized, and no significant changes were seen at *rlen*=600. Therefore, the iteration length (*rlen*) was set at 500.

GSCC: See ***Suppl. Figure S1B*** for monitoring of the progress for the GSCC. Higher iteration lengths than the default was tested from the beginning. At 300 and 500 iterations, the curves were still decreasing but showed signs of a possible plateau. By *rlen*=700, the curve had stabilized, and no further optimization was achieved at *rlen*=900. Therefore, the iteration length (*rlen*) was set at 700.

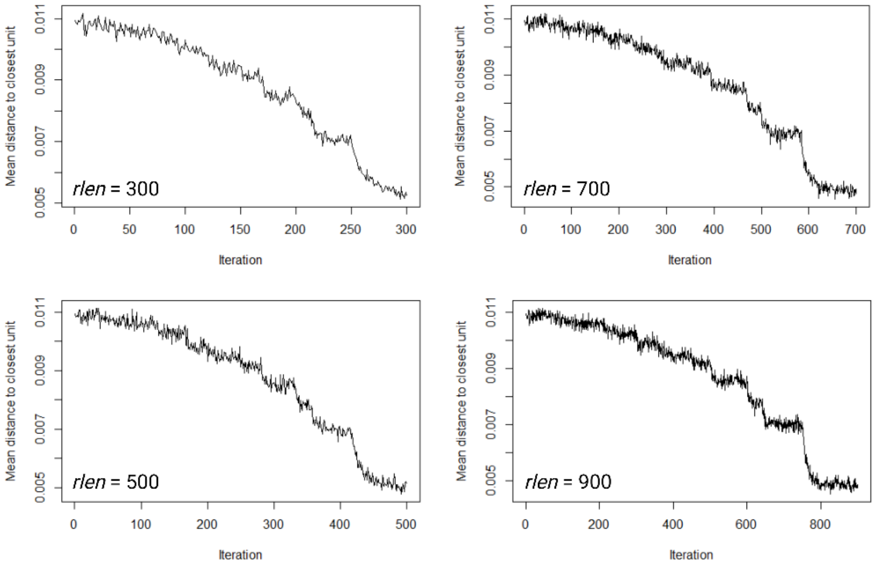

Suppl. Figure S1A: The progress of the mean distance to the closest unit across 100, 200, 300, 400, 500, and 600 iterations for the ECLIPSE dataset. The goal is for the curve to stabilize at a minimum plateau. From the plots, *rlen*=500 appears to be the optimal choice.

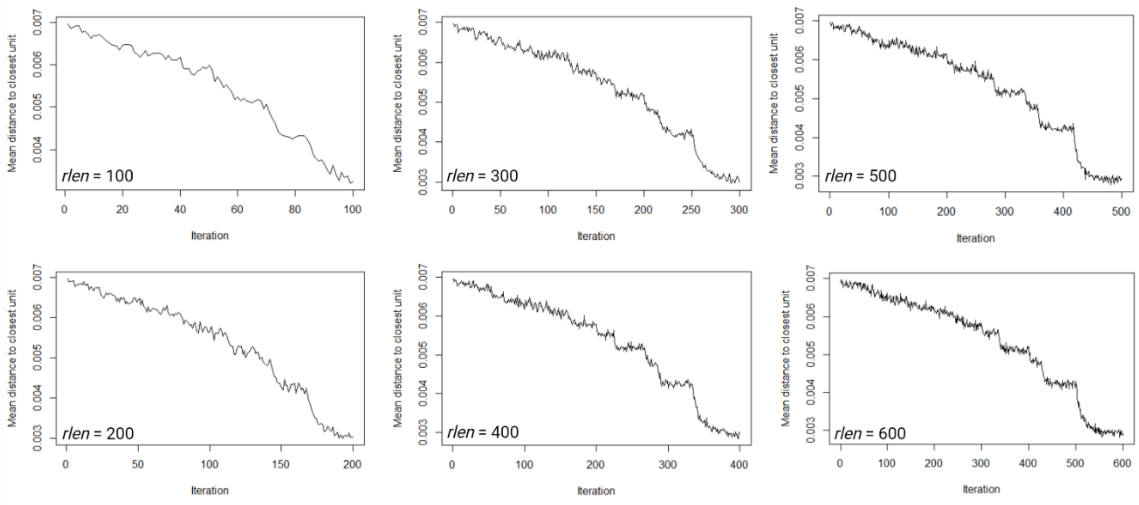

Suppl. Figure S1B: The progress of the mean distance to the closest unit across 300, 500, 700, and 900 iterations for the GSCC dataset. The goal is for the curve to stabilize at a minimum plateau. From the plots, *rlen*=700 appears to be the optimal choice.

2.1.2 Number of clusters optimization

Determining the optimal number of clusters (*k_cluster*) in a dataset is a common challenge in data clustering, often complicated by the ambiguity of the correct choice, which depends on the distribution's shape and scale. When prior knowledge is unavailable, this value must be empirically determined from the data.

A well-established method for this determination in hierarchical clustering involves analyzing the dendrogram's structure.[5] Specifically, one should identify the longest vertical line in the dendrogram that does not intersect any horizontal cluster lines. This vertical line signifies a substantial distance between clusters. By drawing a horizontal line through this longest vertical segment and counting its intersections with horizontal lines representing clusters, the number of intersections provides an estimate of the optimal number of clusters.

ECLIPSE: See ***Suppl. Figure S2A*** for the structure of the dendrogram for the ECLIPSE cohort. The dendrogram indicates that the optimal number of clusters is four. This determination is based on both visual inspection and mathematical calculation, where the longest uncut vertical distance corresponds to a height difference of 4.72 (*15.81 - 11.09*). Subsequent optimal cluster numbers are three and two. Increasing the number of clusters beyond 4 results in more specific but less common comorbidity profiles.

GSCC: See ***Suppl. Figure S2B*** for the structure of the dendrogram for the GSCC. The dendrogram indicates that the optimal number of clusters is three. This determination is based on both visual inspection and mathematical calculation, where the longest uncut vertical distance corresponds to a height difference of 4.4 (*12.11 – 7.71*). The next optimal cluster number is two.

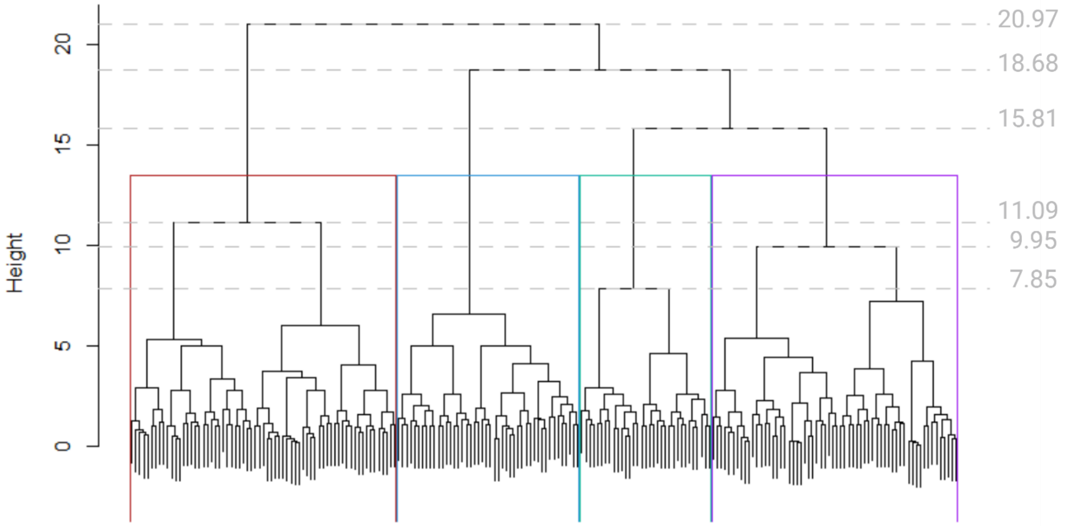

Suppl. Figure S2A: The dendogram's structure of the hierarchical clustering for the ECLIPSE dataset. The goal is to identify the longest vertical line in the dendrogram that does not intersect any horizontal cluster lines. From the dendogram we can see that the longest vertical line is between the horizontal line 3 and 4, meaning that the optimal number of clusters are 4.

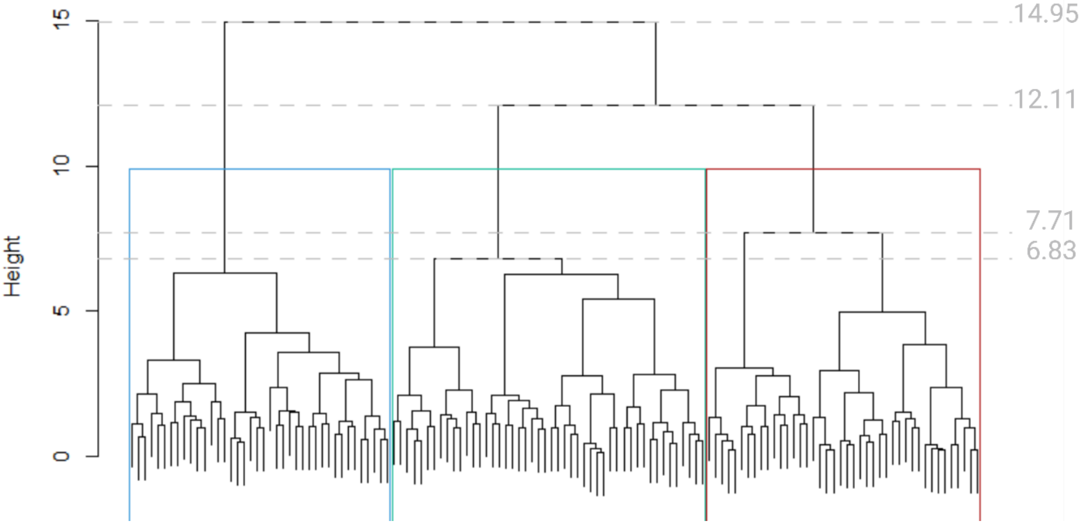

Suppl. Figure S2B: The dendogram's structure of the hierarchical clustering for the GSCC dataset. The goal is to identify the longest vertical line in the dendrogram that does not intersect any horizontal cluster lines. From the dendogram we can see that the longest vertical line is between the horizontal line 2 and 3, meaning that the optimal number of clusters are 3.

2.2 Cluster robustness

Hierarchical clustering is based on a distance-based hierarchy among data points. Unlike *k*-means clustering, increasing the number of clusters (*k*) in hierarchical clustering does not disrupt the composition of previously identified clusters. Instead, it splits existing clusters into

smaller, less descriptive subdivisions. Understanding the order in which clusters split can provide insights into the robustness of each individual cluster.

ECLIPSE: The pipeline discussed in the paper was run with *k* values of 2, 3, 5, and 6, and the results were compared to those obtained with *k=4*.
The musculoskeletal cluster was the first to be identified, indicating it has the easiest to identify comorbidity profile (***Suppl. Figure S3A***). Following this, the mental health cluster was identified, succeeded by the metabolic and circulatory clusters. Notably, as the number of clusters (*k*) increases, the musculoskeletal cluster was the first to split. This resulted in the emergence of a smaller, osteoarthritis-driven cluster, suggesting that osteoarthritis may be influenced by different factors than other musculoskeletal diseases. When *k* was increased to 6, a cluster driven almost exclusively by asthma emerged from the previously identified metabolic cluster. Asthma was the only respiratory disease included in the clustering, implying that its initial placement might have been more incidental rather than indicative of shared underlying pathways.

GSCC: The pipeline discussed in the paper was run with *k* values of 2, 4, and 5, and the results were compared to those obtained with *k=3*.
The mental health cluster was the first to be identified, indicating it has the most easily recognizable comorbidity profile (***Suppl. Figure S3B***). Subsequently, the musculoskeletal cluster and the metabolic/circulatory cluster were identified. As the number of clusters (*k*) increases, the musculoskeletal cluster was the first to split, leading to the emergence of a smaller asthma-driven cluster. This pattern was also observed in the ECLIPSE dataset, suggesting that asthma's placement may be more incidental than indicative of shared underlying pathways. When *k* was increased to 5, a cluster driven almost exclusively by diabetes emerged from the previously identified circulatory/metabolic cluster. The GSCC had a significantly lower number of patients with metabolic diseases compared to the ECLIPSE dataset, and this split could thus indicate a possible separation into distinct metabolic and circulatory clusters.

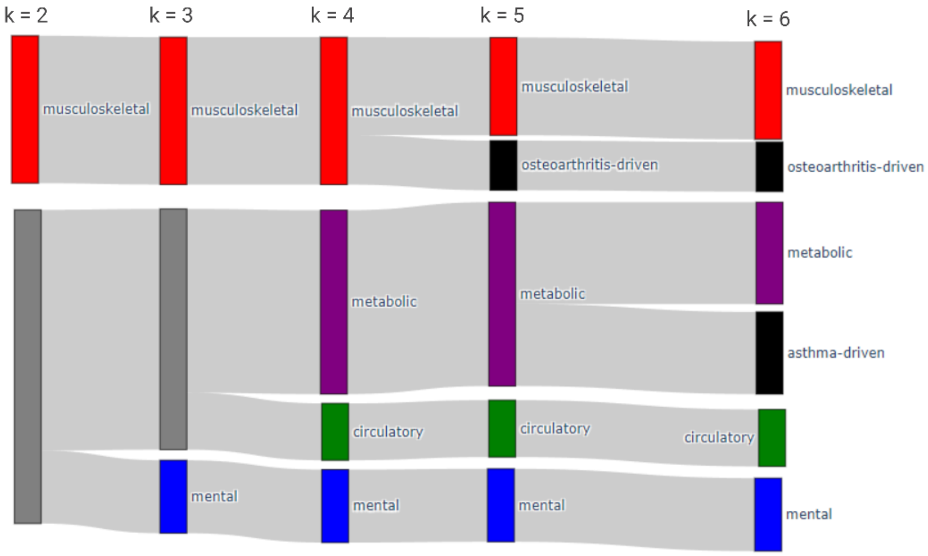

Suppl. Figure S3A: A Sankey plot illustrating the order of cluster identification and their subsequent splits across different values of *k* in the ECLIPSE dataset. The sequence of cluster identification is as follows: musculoskeletal, mental, and then metabolic or circulatory. The plot also demonstrates that increasing the number of clusters (*k*) from the optimal four to six results in the emergence of two additional clusters: one driven by asthma and the other by osteoarthritis.

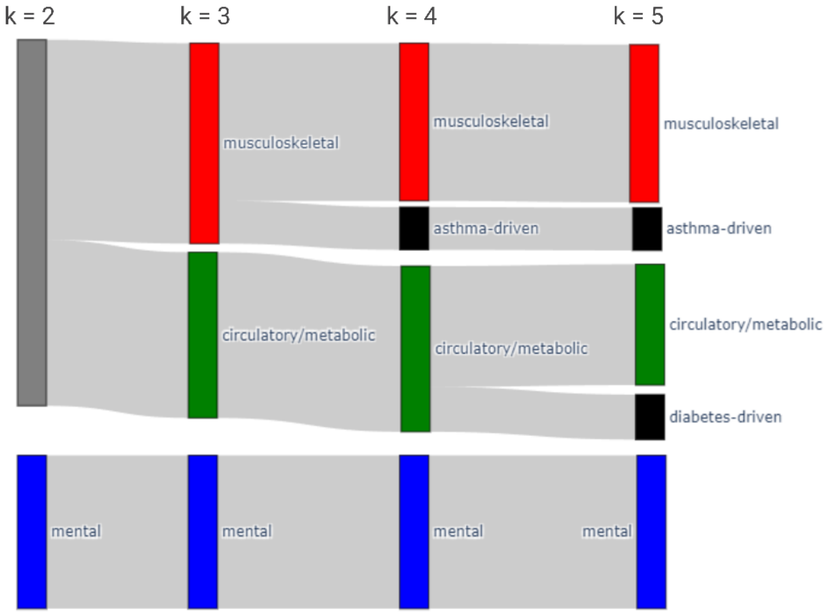

Suppl. Figure S3B: A Sankey plot illustrating the order of cluster identification and their subsequent splits across different values of *k* in the GSCC dataset. The sequence of cluster identification is as follows: mental and then metabolic/ circulatory or musculoskeletal. The plot also demonstrates that increasing the number of clusters (*k*) from the optimal three to five results in the emergence of two additional clusters: one driven by asthma and the other by diabetes.

2.3 Cluster interpretation

When using SOMs, each patient is positioned on a map according to their specific comorbidity profile. However, these maps do not capture the primary drivers behind each cluster's formation. To identify these drivers, we trained random forest (RF) models[6] —a supervised machine learning method—optimized through grid-search on the labeled clusters using a one-against-the-rest approach. After training the models, we employed the SHapley Additive exPlanations (SHAP) method[7] to estimate the importance magnitudes of input features. SHAP values provide an estimate of feature relevance in predicting cluster labels, offering valuable insights for further cluster interpretation.

ECLIPSE: In the ECLIPSE cohort, four clusters were identified. As mentioned in *section 2.2*, the order in which they were identified was musculoskeletal, mental, and then metabolic or circulatory. Some of these trends are also evident from the decisions seen in the SHAP beeswarm plots (***Suppl. Figure S4A***). The musculoskeletal cluster, as expected, shows that the decision to form this cluster was primarily based on the presence of muscle wasting (highest global importance +0.21, ***Suppl. Figure S4B***), osteoarthritis, and osteoporosis, as well as the absence of obesity, making it easy for both the SOMs and the RF models to identify. Following this, the mental cluster was identified, primarily based on the presence of depression (highest global importance +0.16) and anxiety, and the absence of muscle wasting. The absence of muscle wasting might initially seem counterintuitive, but it is explained by the fact that patients with muscle wasting were already mainly placed in the first identified cluster, the musculoskeletal one. Afterward, either the circulatory or the metabolic cluster was identified. The decisions for the circulatory cluster were based on the presence of peptic ulcer (highest global importance +0.1), heart failure, and heart attack, indicating that peptic ulcer was the primary driver of this cluster. This finding, though not explored further in the study, could suggest shared pathways between cardiovascular disease and peptic ulcer. Lastly, the metabolic cluster was primarily driven by the presence of obesity (highest global importance +0.13) and the absence of muscle wasting (highest global importance +0.12).

GSCC: In the GSCC, three clusters were identified. As mentioned in *section 2.2*, the order in which they were identified was mental, followed by metabolic/circulatory or musculoskeletal.

Some of these trends are also evident from the decisions seen in the SHAP beeswarm plots (***Suppl. Figure S5A***). The mental cluster shows that the decision to form this cluster was almost solely based on the presence of depression (highest global importance +0.3, ***Suppl. Figure S5B***). The decision to form the metabolic/circulatory cluster was primarily based on the presence of hypertension (highest global importance +0.21) and the absence of depression. The absence of depression in this cluster can be explained by the fact that these patients were already placed in the first identified cluster, the mental one. Lastly, the formation of the musculoskeletal cluster seems to be more of a "what is left" scenario, as it was based on the absence of both depression and hypertension (highest global importance +0.16 and +0.2, respectively), making it the weakest identified cluster in the GSCC dataset.

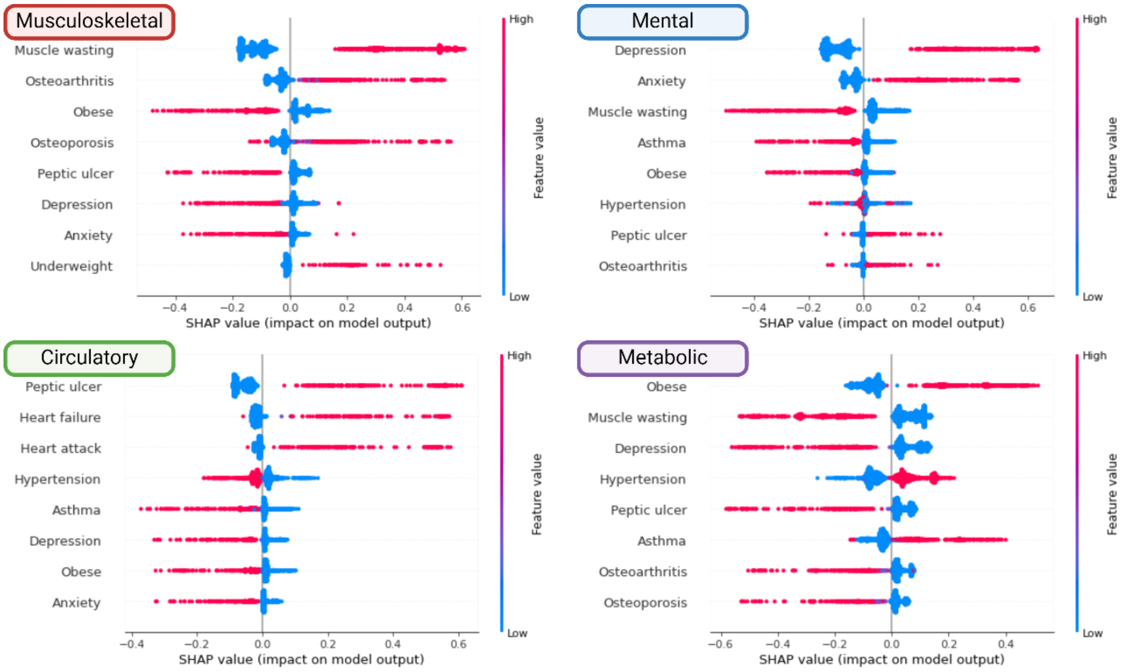

Suppl. Figure S4A: Impact of comorbidity prevalence in the ECLIPSE dataset on the random forest models decisions. The top 10 most impactful comorbidities are presented in descending order of importance, with the most significant ones placed at the top. Each dot within each row represents an individual patient's data. The x-axis displays the SHAP values, indicating the magnitude and direction of impact (positive values signify an increased probability of belonging to the cluster). The color scale on the right represents the binary comorbidity values, with red indicating presence and blue indicating absence. The musculoskeletal cluster is primarily driven by the presence of muscle wasting and osteoarthritis, and the absence of obesity. The mental cluster is primarily driven by the presence of depression and anxiety, and the absence of muscle wasting. The circulatory cluster is primarily driven by the presence of peptic ulcer, heart failure, and heart attack. The metabolic cluster is primarily driven by the presence of obesity, and the absence of muscle wasting and depression.

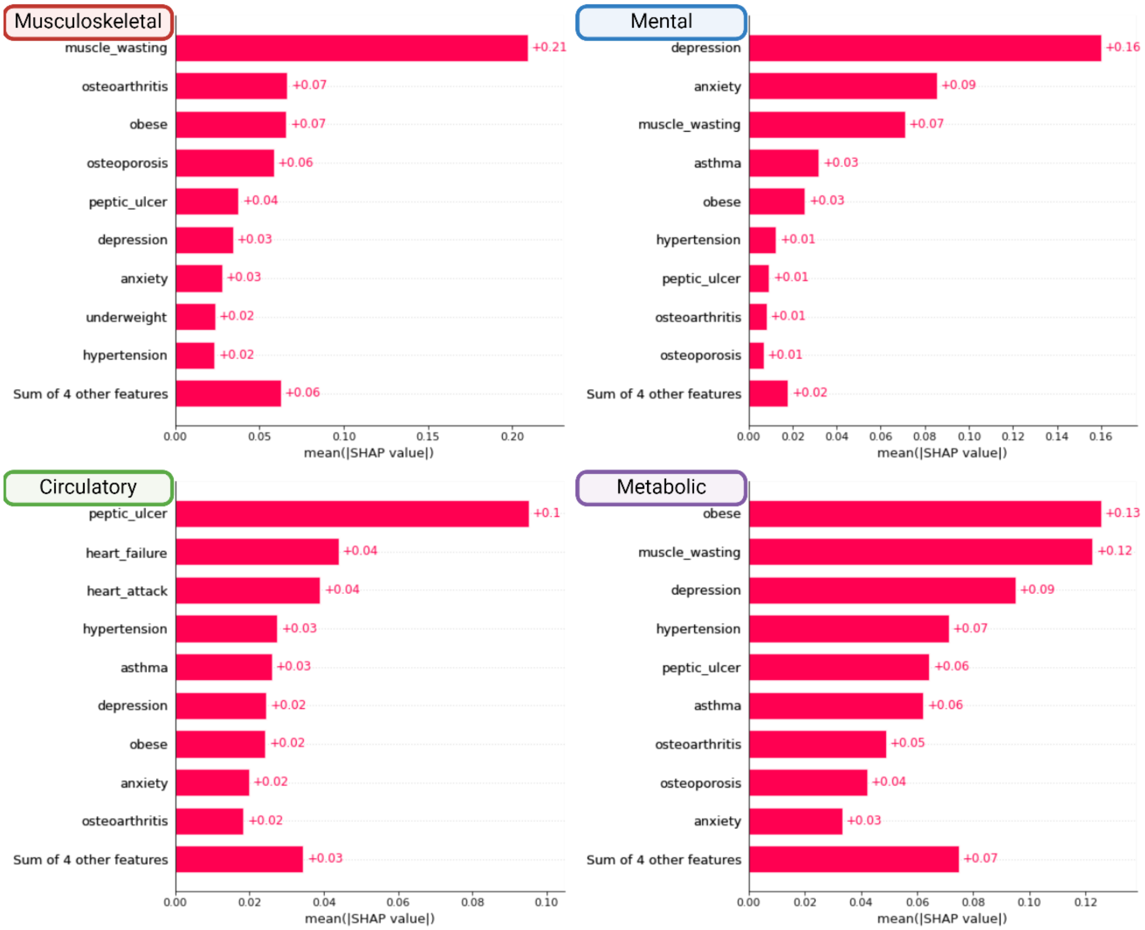

Suppl. Figure S4B: Global importance of each comorbidity in the ECLIPSE dataset on the random forest models decisions. The global importance of a comorbidity is determined by calculating the mean absolute value for that comorbidity across all the given samples. The musculoskeletal cluster is primarily impacted by muscle wasting. The mental cluster is primarily impacted by depression. The circulatory cluster is primarily impacted by peptic ulcer. The metabolic cluster is primarily impacted by obesity and muscle wasting.

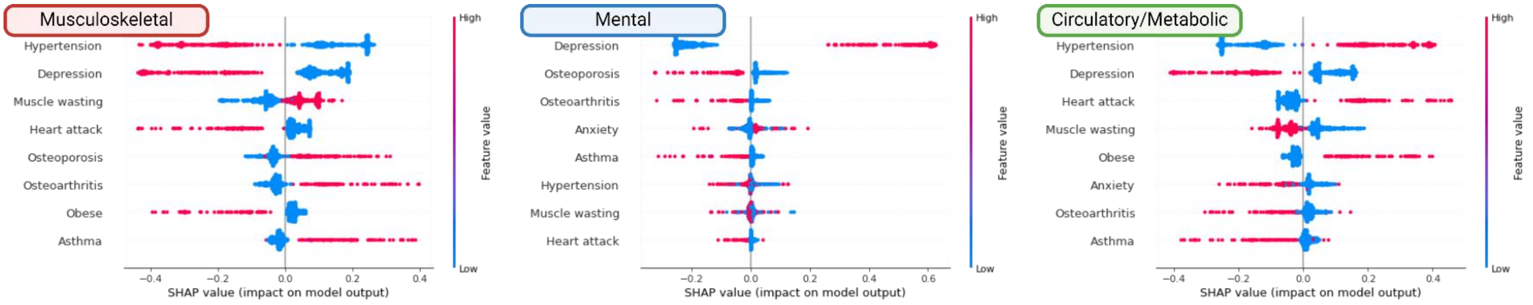

Suppl. Figure S5A: Impact of comorbidity prevalence in the GSCC dataset on the random forest models decisions. The top 10 most impactful comorbidities are presented in descending order of importance, with the most significant ones placed at the top. Each dot within each row represents an individual patient's data. The x-axis displays the SHAP values, indicating the magnitude and direction of impact (positive values signify an increased probability of belonging to the cluster). The color scale on the right represents the binary comorbidity values, with red indicating presence and blue indicating absence. The musculoskeletal cluster is primarily driven by the presence of muscle wasting, and the absence of hypertension and depression. The mental cluster is primarily driven by the presence of depression. The circulatory/metabolic cluster is primarily driven by the

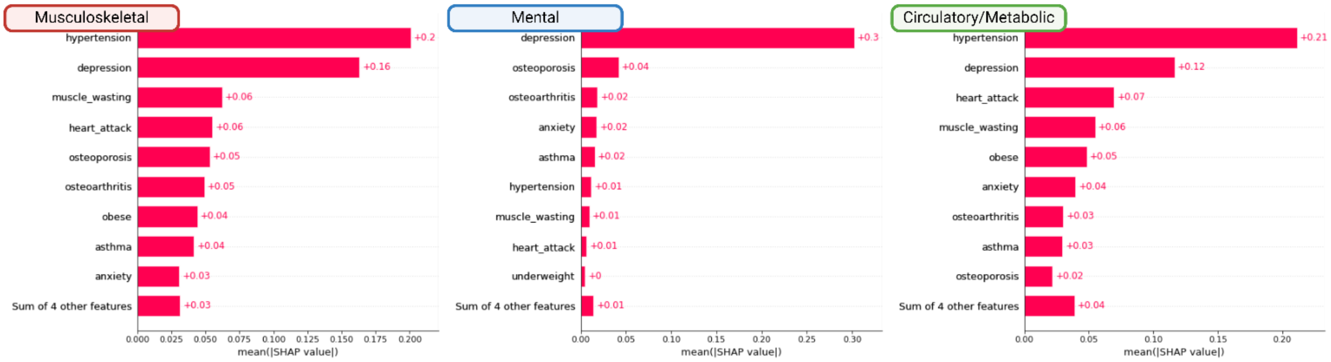

presence of hypertension, heart attack, and obesity, and the absence of depression and muscle wasting.

Suppl. Figure S5B: Global importance of each comorbidity in the GSCC dataset on the random forest models decisions. The global importance of a comorbidity is determined by calculating the mean absolute value for that comorbidity across all the given samples. The musculoskeletal cluster is primarily impacted by hypertension and depression. The mental cluster is primarily impacted by depression. The circulatory/metabolic cluster is primarily impacted by hypertension.

2.4 Multiple cluster qualifications

Hierarchical clustering groups patients by merging those with similar traits first, gradually forming broader or meaningful subgroups. This nested structure assigns each patient to only one cluster, favoring clear distinctions but potentially overlooking finer nuances. For instance, in ECLIPSE, 448 comorbidity combinations are condensed into 4 subgroups, while in GSCC, 237 combinations are reduced to 3 subgroups. In this way, clustering helps simplify complex data, moving beyond the traditional "one-size-fits-all" approach.

However, some patients could qualify for multiple subgroups; the method resolves this by assigning the patient to the most similar cluster, based on the algorithms' decisions described in *sections 2.2* and *2.3*. To illustrate how the algorithm handles some of these cases, upset plots (a technique to visualize set intersections based on grouping) have been generated for the top 15 intersections between two and three combinations for both ECLIPSE (***Suppl. Figure S6A/B***) and GSCC (***Suppl. Figure S7A/B***).

ECLIPSE: In ***Suppl. Figure S6A***, we see the top 15 intersections in patients with two comorbidities. Nine of these intersections span two comorbidity categories, meaning the patients qualify for two clusters. The algorithm resolves this in a "rock-paper-scissors" manner, following the hierarchy described in *section 2.2*, where the most robust cluster determines the patient's final assignment. Musculoskeletal beats mental, mental beats circulatory, circulatory beats metabolic, and metabolic beats other (asthma and peptic ulcer). One exception is observed in the intersection between obesity (*purple; metabolic*) and peptic ulcer (*light grey; other*), where patients are assigned to the circulatory cluster. This is due to the strong link between peptic ulcer and the circulatory cluster, as shown in ***Figure 3*** and ***Suppl. Figure S4A***, despite peptic ulcer being a distinct disease category. Although not explored further in this study, this finding may suggest shared pathways between cardiovascular disease and peptic ulcer.

In ***Suppl. Figure S6B***, we see the top 15 intersections in patients with three comorbidities. 14 of these intersections span up to three comorbidity categories, meaning the patients qualify for multiple clusters. The algorithm generally follows a "majority rules" approach, where if two comorbidities belong to the same category, the patient is assigned to the corresponding

cluster. Asthma is linked to the metabolic cluster, as shown in ***Figure 3*** and ***Suppl. Figure S4A***, and is treated as a metabolic disease by the algorithm. If patients belong to three categories, the hierarchy from *section 2.2* is applied. However, there is one exception to this rule: hypertension acts as a universal "joker" (also seen in ***Figure 3***, found in all clusters), and in most cases, it triggers the application of the rule from *section 2.2*.

GSCC: In ***Suppl. Figure S7A***, we see the top 15 intersections in patients with two comorbidities. Eight of these intersections span two comorbidity categories, meaning the patients qualify for two clusters. While ECLIPSE's division was more focused on multiple comorbidities, following the hierarchy outlined in *section 2.2*, the GGSC algorithm is more focused on single diseases. The three clusters are primarily driven by one disease from each category, as shown in ***Figure 3*** and ***Suppl. Figure S5A***. Muscle wasting drives the musculoskeletal cluster, depression drives the mental cluster, and hypertension drives the combined circulatory-metabolic cluster. Thus, these three comorbidities determine a patient's assignment if they qualify for multiple categories.

In ***Suppl. Figure S7B***, we see the top 15 intersections in patients with three comorbidities. 12 of these intersections span up to three comorbidity categories, meaning the patients qualify for multiple clusters. The algorithm generally follows a "majority rules" approach, where if two comorbidities belong to the same category, the patient is assigned to the corresponding cluster. If patients belong to three categories, the assignment becomes disease-specific: if hypertension is present, the patient is assigned to the circulatory-metabolic cluster; if muscle wasting is present, the patient is assigned to the musculoskeletal cluster; and if depression is present, the patient is assigned to the mental cluster.

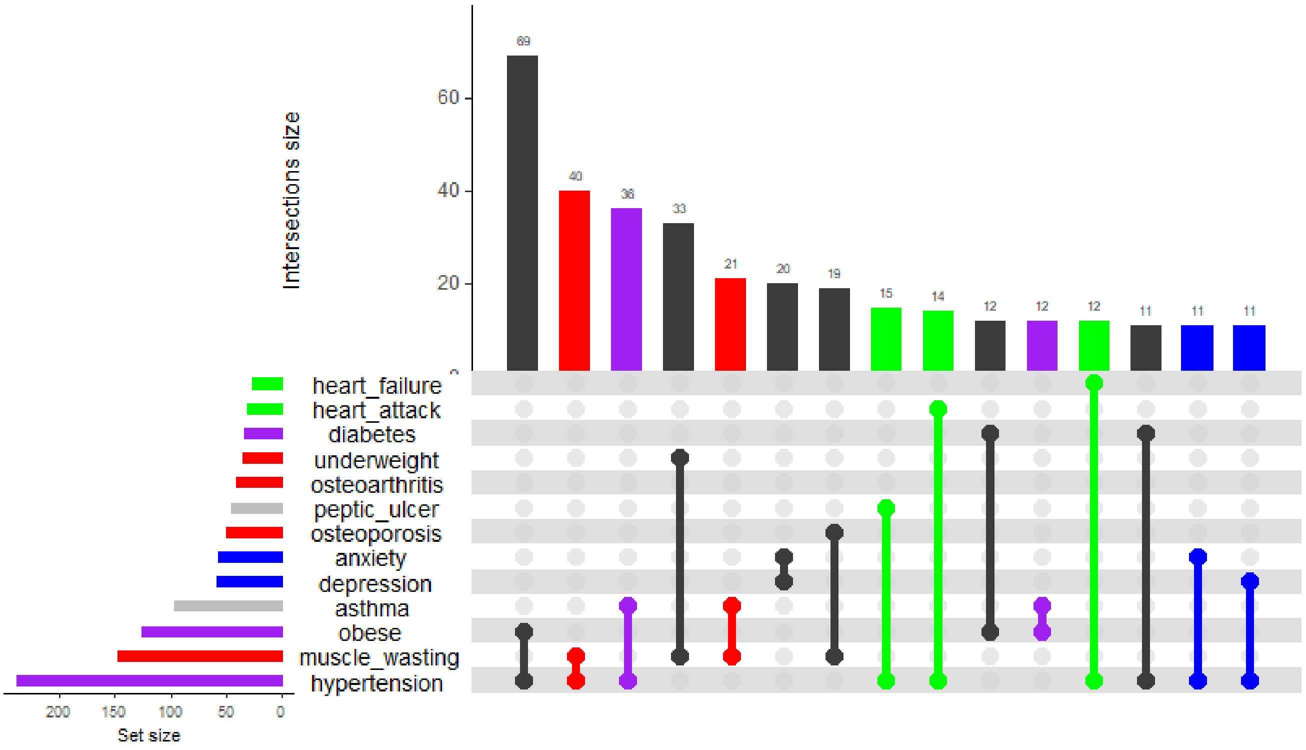

Suppl. Figure S6A: Upset plot showing the top 15 intersections for the ECLIPSE cohort, focusing on patients with exactly two comorbidities. The rows of the matrix correspond to the comorbidities, while the columns represent the intersections between them. Bar charts display the number of comorbidities (*left*) and intersections (*top*). If an intersection and its corresponding bar chart (*top*) are highlighted in color, it indicates that the intersection spans multiple comorbidity categories, with the color denoting the final cluster assignment for patients with those specific intersections. Most assignments follow the general hierarchy described in *section 2.2*: musculoskeletal > mental > circulatory > metabolic > other. Color meanings: Red = Musculoskeletal; Blue = Mental; Green = Circulatory; Purple = Metabolic; Light grey = Other (asthma and peptic ulcer).

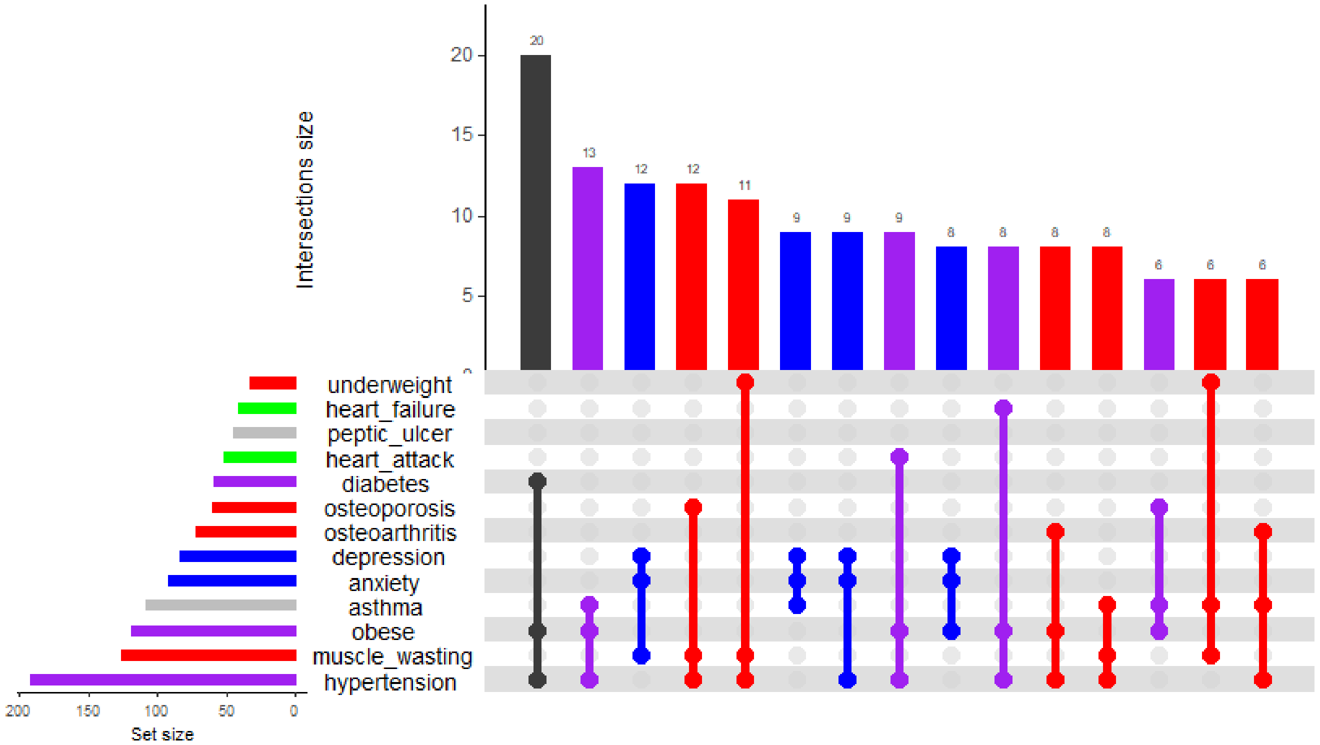

Suppl. Figure S6B: Upset plot showing the top 15 intersections for the ECLIPSE cohort, focusing on patients with exactly three comorbidities. The rows of the matrix correspond to the comorbidities, while the columns represent the intersections between them. Bar charts display the number of comorbidities (*left*) and intersections (*top*). If an intersection and its corresponding bar chart (*top*) are highlighted in color, it indicates that the intersection spans multiple comorbidity categories, with the color denoting the final cluster assignment for patients with those specific intersections. Most assignments follow a "majority rules" approach, where if two comorbidities belong to the same category, the patient is assigned to the corresponding cluster. Color meanings: Red = Musculoskeletal; Blue = Mental; Green = Circulatory; Purple = Metabolic; Light grey = Other (asthma and peptic ulcer).

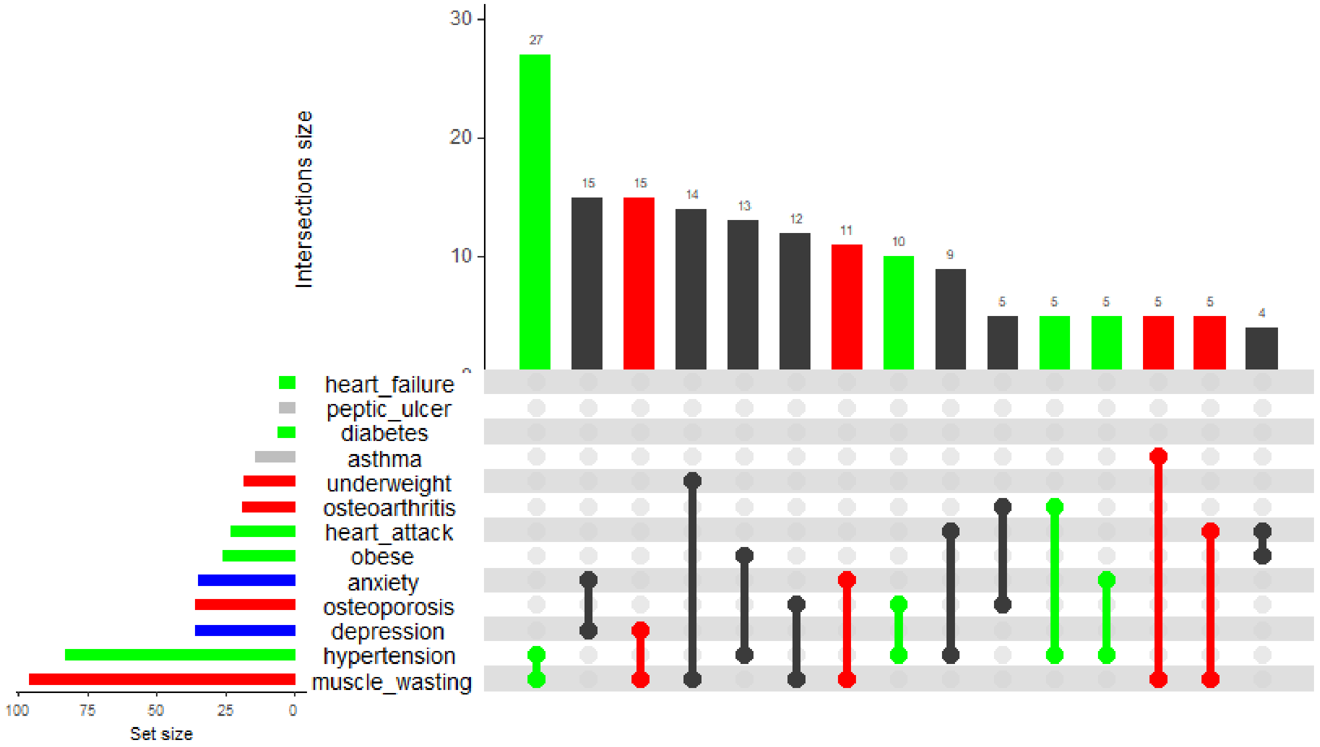

Suppl. Figure S7A: Upset plot showing the top 15 intersections for the GSCC cohort, focusing on patients with exactly two comorbidities. The rows of the matrix correspond to the comorbidities, while the columns represent the intersections between them. Bar charts display the number of comorbidities (left) and intersections (top). If an intersection and its corresponding bar chart (top) are highlighted in color, it indicates that the intersection spans multiple comorbidity categories, with the color denoting the final cluster assignment for patients with those specific intersections The algorithm is primarily driven by specific diseases rather than a strict order. Hypertension (green; Circulatory-Metabolic) and muscle wasting (red; Musculoskeletal) appear in most intersections across disease categories, and in these cases, they determine the patient's cluster, with hypertension having a stronger influence than muscle wasting. Color meanings: Red = Musculoskeletal; Blue = Mental; Green = Circulatory-Metabolic; Light grey = Other (asthma and peptic ulcer).

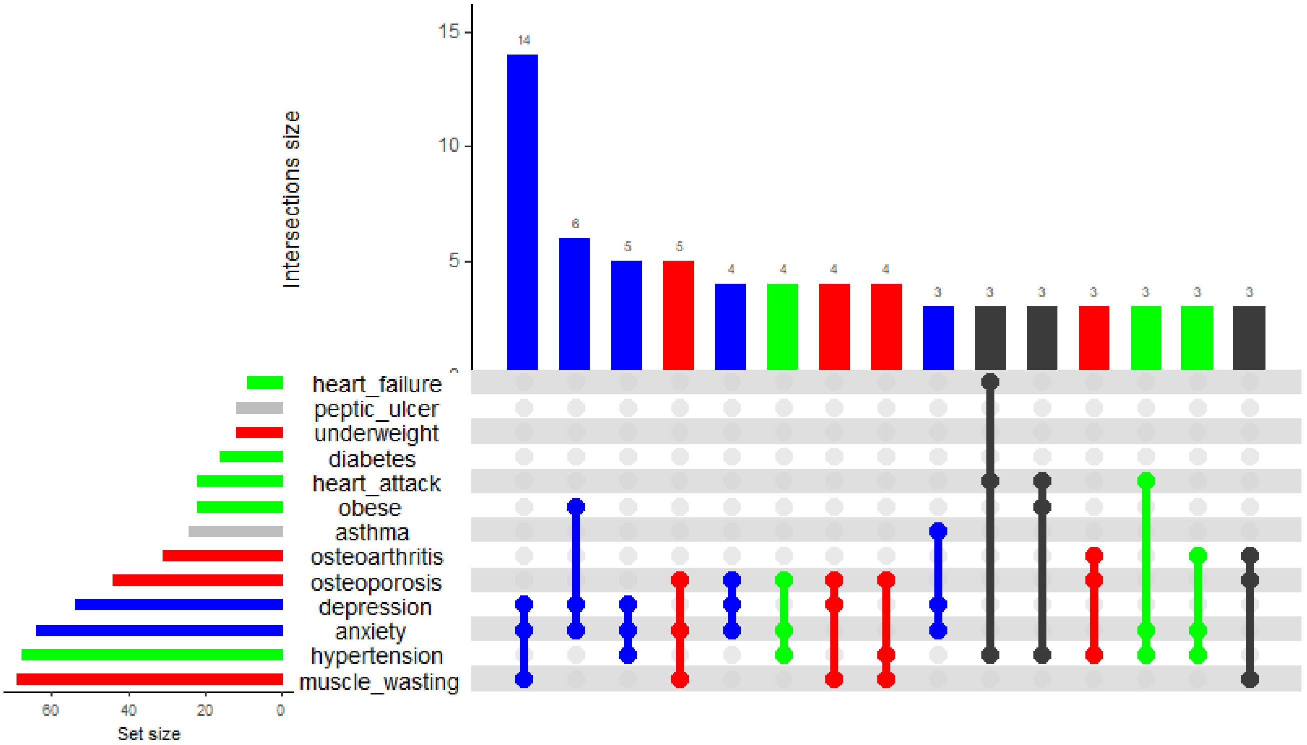

Suppl. Figure S7B: Upset plot showing the top 15 intersections for the GSCC cohort, focusing on patients with exactly three comorbidities. The rows of the matrix correspond to the comorbidities, while the columns represent the intersections between them. Bar charts display the number of comorbidities (left) and intersections (top). If an intersection and its corresponding bar chart (top) are highlighted in color, it indicates that the intersection spans multiple comorbidity categories, with the color denoting the final cluster assignment for patients with those specific intersections. Most assignments follow a "majority rules" approach, where if two comorbidities belong to the same category, the patient is assigned to the corresponding cluster. Color meanings: Red = Musculoskeletal; Blue = Mental; Green = Circulatory-Metabolic; Light grey = Other (asthma and peptic ulcer).

Section 3: Testing the proportional hazards assumption

The Cox proportional hazards model operates under the assumption that the hazard ratios between different levels of the covariates remain constant over time, known as the proportional hazards (PH) assumption. To validate this model, it is essential to perform tests to determine whether the PH assumption holds. One commonly used test for this purpose is the cumulative martingale residuals test.[8] Subsequently, we can employ graphical procedures based on simulated score processes, assuming the Cox model's correctness, to identify potential reasons and locations where the PH assumption might be violated.

3.1 ECLIPSE

3.1.1 Univariate PH model

The cumulative martingale residuals test did not reject the proportional hazards assumption (***Suppl. Table S2A***). The plots verify the conclusion based on the p-value (***Suppl. Figure S8A***) however a small downward trend is seen for the mental cluster within the first ~350 days.

Suppl. Table S2A: Using the cumulative martingale residuals test to assess the proportional hazards assumption in a univariate model on the ECLIPSE dataset, we observe that the p-value is high (>0.05). Consequently, we cannot reject the null hypothesis that the proportional hazards assumption is fulfilled.

Cluster	p-values
Cluster 1	0.067
Cluster 2	0.054
Cluster 3	0.253
Cluster 4	0.462
Cluster 1 = Musculoskeletal; Cluster 2 = Mental, Cluster 3 = Circulatory; Cluster 4 = Metabolic	

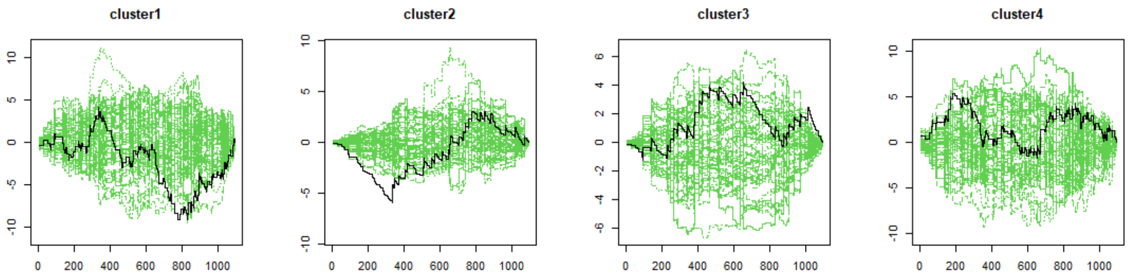

Suppl. Figure S8A: By employing graphical procedures based on 50 simulated score processes (green curves), we can compare them to the observed score process (black curve). If the proportional hazards assumption is fulfilled, the black curve should look like one of the green curves. cluster1 = Musculoskeletal; cluster2 = Mental, cluster3 = Circulatory; cluster4 = Metabolic.

3.1.2 Multivariate PH model

In the adjustment of the Cox PH model, the cumulative martingale residuals test did not reject the PH assumption (***Suppl. Table S2B***). This conclusion is further supported by the plots (***Suppl. Figure S8B***), where the mental cluster shows only a slight but statistically insignificant decrease at the beginning.

Suppl. Table S2B: Using the cumulative martingale residuals test to assess the proportional hazards assumption in the multivariate model on the ECLIPSE dataset, we observe that the p-value is high (>0.05). Consequently, we cannot reject the null hypothesis that the proportional hazards assumption is fulfilled.

Cluster	p-values
Cluster 1	0.100
Cluster 2	0.261
Cluster 3	0.194
Cluster 4	0.559
Age	0.748
Sex	0.110
BMI	0.461
%FEV_1	0.683
Emphysema	0.204

Cluster 1 = Musculoskeletal; Cluster 2 = Mental, Cluster 3 = Circulatory; Cluster 4 = Metabolic; BMI = Body mass index; %FEV_1 = percentage predicted forced expiratory volume in 1 second; Emphysema = -950 Hounsfield Units

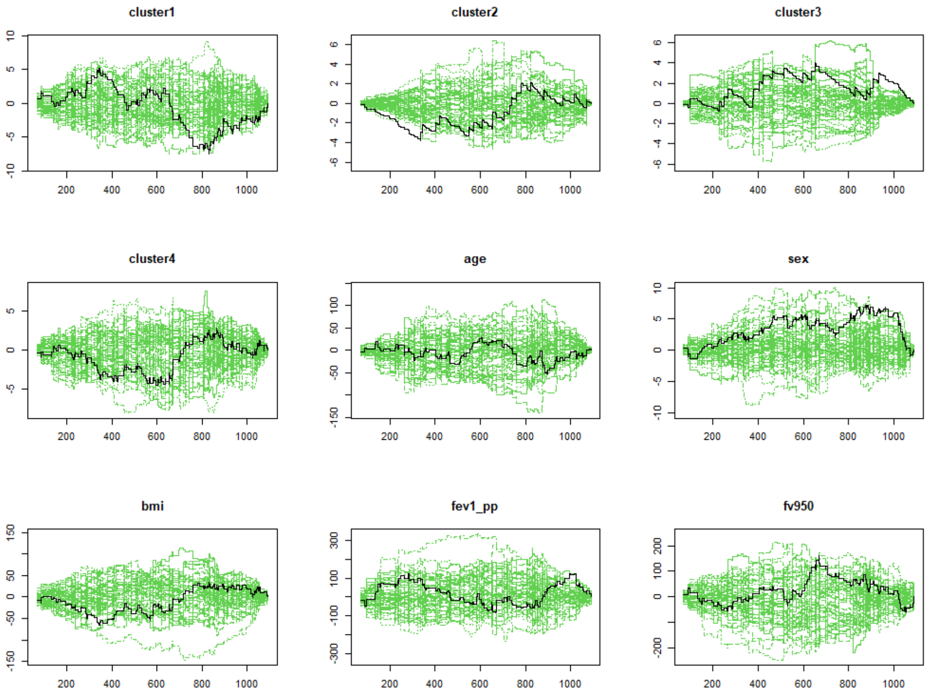

Suppl. Figure S8B: By employing graphical procedures based on 50 simulated score processes (green curves), we can compare them to the observed score process (black curve). cluster1 = Musculoskeletal; cluster2 = Mental, cluster3 = Circulatory; cluster4 = Metabolic; bmi = body mass index, fev1_pp = percentage predicted forced expiratory volume in 1 second; fv950 = emphysema extent (-950 Hounsfield Units)

3.2 GSCC

3.2.1 Univariate PH model

The cumulative martingale residuals test did not reject the proportional hazards assumption (***Suppl. Table S3A***). The plots verify the conclusion based on the p-value (***Suppl. Figure S9A***).

Suppl. Table S3A: Using the cumulative martingale residuals test to assess the proportional hazards assumption in a univariate model on the GSCC dataset, we observe that the p-value is high (>0.05). Consequently, we cannot reject the null hypothesis that the proportional hazards assumption is fulfilled.

Cluster	p-values
Cluster 1	0.392
Cluster 2	0.161
Cluster 3	0.197
Cluster 1 = Musculoskeletal; Cluster 2 = Mental, Cluster 3 = Circulatory/Metabolic	

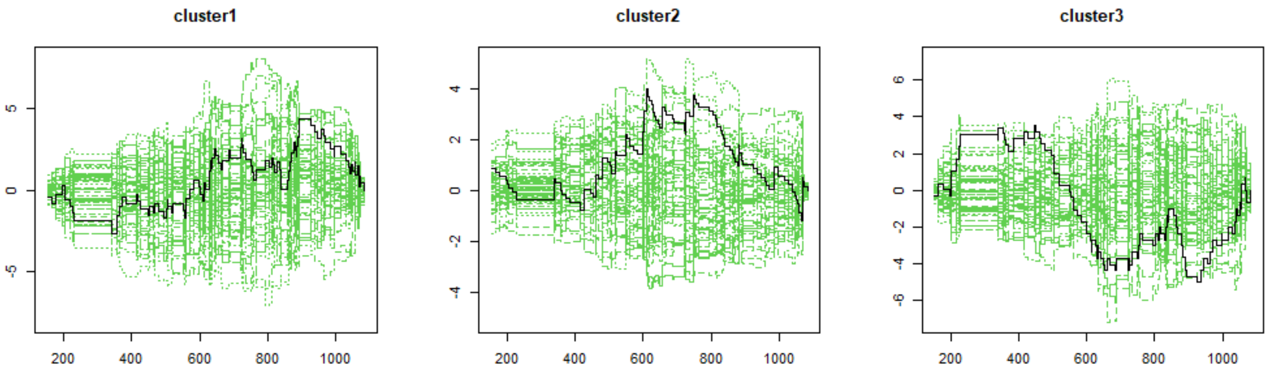

Suppl. Figure S9A: By employing graphical procedures based on 50 simulated score processes (green curves), we can compare them to the observed score process (black curve). If the proportional hazards assumption is fulfilled, the black curve should look like one of the green curves. cluster1 = Musculoskeletal; cluster2 = Mental, cluster3 = Circulatory/Metabolic.

3.2.2 Multivariate PH model

When doing multivariate adjustment (age, sex, BMI, FEV_1 and emphysema extent) of the Cox PH model, the cumulative martingale residuals test did still not reject the PH assumption (***Suppl. Table S3B***). This conclusion is further supported by the plots (***Suppl. Figure S9B).***

Suppl. Table S3B: Using the cumulative martingale residuals test to assess the proportional hazards assumption in the multivariate model on the GSCC dataset, we observe that the p-value is high (>0.05). Consequently, we cannot reject the null hypothesis that the proportional hazards assumption is fulfilled.

Cluster	p-values
Cluster 1	0.366
Cluster 2	0.354
Cluster 3	0.242
Age	0.496
Sex	0.406
BMI	0.749
$\%FEV_1$	0.908
Emphysema	0.531

Cluster 1 = Musculoskeletal; Cluster 2 = Mental, Cluster 3 = Circulatory/Metabolic; BMI = Body mass index; $\%FEV_1$ = percentage predicted forced expiratory volume in 1 second; Emphysema = -950 Hounsfield Units

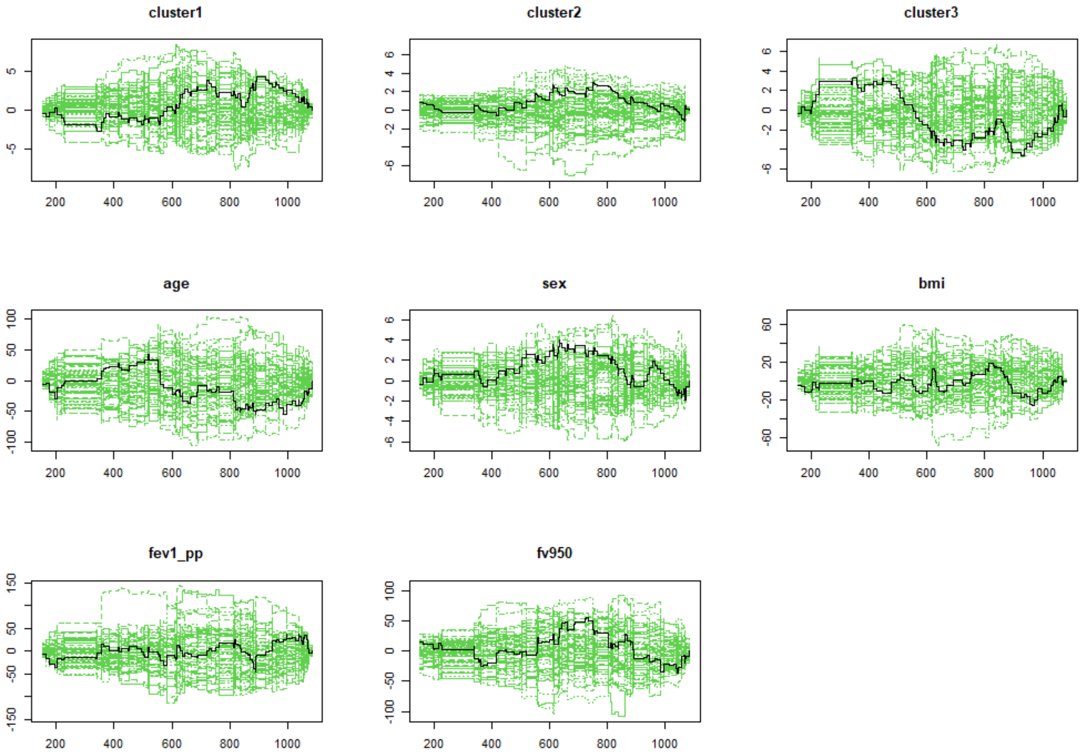

Suppl. Figure S9B: By employing graphical procedures based on 50 simulated score processes (green curves), we can compare them to the observed score process (black curve). cluster1 = Musculoskeletal; cluster2 = Mental, cluster3 = Circulatory/Metabolic; bmi = body mass index, fev1_pp = percentage predicted forced expiratory volume in 1 second; fv950 = emphysema extent (-950 Hounsfield Units)

Section 4: Time-to-first-severe-exacerbation

Methods: In ECLIPSE, outcome data on acute exacerbations of COPD (AECOPD) were collected through monthly phone calls. The start date, end date, and severity of each exacerbation were recorded as mild (no treatment or hospitalization), moderate (requiring medication), or severe (requiring hospitalization) throughout the study period. Time-to-event analyses were performed using Kaplan-Meier curves (***Suppl. Figure S10***) and Cox proportional hazards models (***Suppl. Table S4***). Results are shown in both univariate and covariate-adjusted forms, with adjustments for age, sex, BMI, percentage predicted FEV1 (FEV1%), and emphysema extent.

Results: Significant differences in unadjusted severe AECOPD risk were found between clusters (log-rank $p < 0.0001$; ***Suppl. Figure S10***). The musculoskeletal cluster had a significantly higher risk (hazard ratio (HR) 1.72; 95% CI 1.31-2.24; $p < 0.0001$; ***Suppl. Table S4***) compared to patients without comorbidities. The mental cluster also showed increased risk (HR 1.41; 95% CI 1.03-1.91; $p = 0.03$). After covariate adjustment, the musculoskeletal cluster remained significant (HR 1.40; 95% CI 1.03-1.89; $p = 0.03$), while the mental cluster's increased risk diminished. Replication was not possible in GSCC due to unavailable data.

Conclusion: The musculoskeletal cluster was linked to a higher risk of severe AECOPD requiring hospitalization, consistent with trends in ***Table 2***. Patients in the musculoskeletal and mental clusters had higher prior AECOPD rates than average, suggesting a need for targeted interventions. Current treatments for these groups may be inadequate.

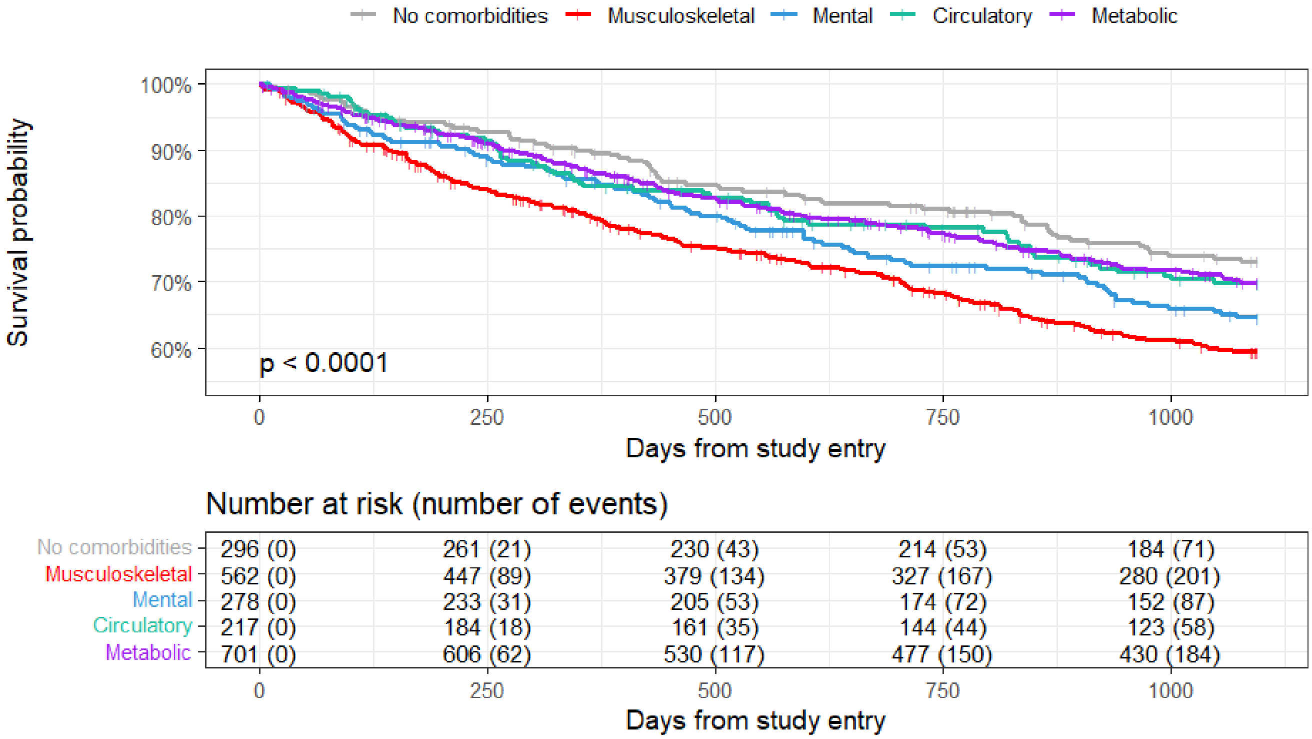

Suppl. Figure S10: Kaplan-Meier curves showing time-to-first-severe-exacerbation (requiring hospitalization) over 1,095 days (3 years) in the reference group without comorbidities and the four clusters across ECLIPSE. Patients who left the study before 1,095 days were censored.

Suppl. Table S4: Cox proportional hazards (PH) models of risk of severe exacerbations in the ECLIPSE cohort. Patients with no comorbidities were used as reference. Data are presented as hazard ratios with 95% confidence intervals and p-values. Multivariate mortality analyses are adjusted for age, sex, body mass index at baseline, percentage predicted forced expiratory volume in 1 second and emphysema extent (-950 Hounsfield Units).

	C1: Musculoskeletal	C2: Mental	C3: Circulatory	C4: Metabolic
**Univariate**	1.72 (1.31-2.24): p<0.0001	1.41 (1.03-1.91): p=0.03	1.16 (0.82-1.63): p=0.40	1.16 (0.88-1.51): p=0.29
**Multivariate**	1.40 (1.03-1.89): p=0.03	1.29 (0.92-1.80): p=0.14	0.93 (0.62-1.38): p=0.70	1.17 (0.86-1.58): p=0.32

Section 5: The frequencies of comorbidities identified in each cluster in both the Evaluation of COPD Longitudinally to Identify Predictive Surrogate Endpoints (ECLIPSE) cohort and the Groningen Severe COPD Cohort (GSCC)

Suppl. Table S5: The frequencies of comorbidities identified in each cluster from the ECLIPSE cohort. The columns represent the clusters, and the rows indicate the number of patients with that comorbidity and the prevalence frequency (%) of the comorbidities within that respective cluster.

**Comorbidities**	**Cluster 1:** Musculoskeletal	**Cluster 2:** Mental	**Cluster 3:** Circulatory	**Cluster 4:** Metabolic	**p-value**
N	*562*	*278*	*217*	701	
Anxiety, n (%)	69 (12.3)[£]	193 (69.4)[†]	9 (4.1)[£]	67 (9.6)[£]	<0.001
Asthma, n (%)	151 (27.3)	45 (16.3)[£]	14 (6.5)[£]	231 (33.3)[†]	<0.001
Depression, n (%)	72 (12.8)[£]	220 (79.4)[†]	3 (1.4)[£]	40 (5.7)[£]	<0.001
Diabetes, n (%)	17 (3.0)[£]	23 (8.3)	33 (15.3)	134 (19.3)[†]	<0.001
Heart attack, n (%)	25 (4.5)[£]	20 (7.2)	77 (35.0)[†]	49 (7.0)[£]	<0.001
Heart failure, n (%)	11 (2.0)[£]	11 (4.0)[£]	72 (33.2)[†]	42 (6.0)[£]	<0.001
Hypertension, n (%)	174 (31.3)[£]	117 (42.1)	81 (37.3)[£]	454 (65.3)[†]	<0.001
Muscle wasting, n (%)	434 (77.2)[†]	33 (11.9)[£]	38 (17.5)[£]	6 (0.9)[£]	<0.001
Obesity, n (%)	12 (2.1)[£]	60 (21.6)[£]	27 (12.4)[£]	372 (53.1)[†]	<0.001
Osteoarthritis, n (%)	134 (23.8)[†]	79 (28.4)[†]	4 (1.8)[£]	64 (9.1)[£]	<0.001
Osteoporosis, n (%)	161 (28.6)[†]	54 (19.4)[†]	15 (6.9)[£]	43 (6.1)[£]	<0.001
Peptic ulcer, n (%)	26 (4.6)[£]	55 (19.8)[†]	110 (50.9)[†]	18 (2.6)[£]	<0.001
Underweight, n (%)	97 (17.3)[†]	6 (2.2)[£]	2 (0.9)[£]	1 (0.1)[£]	<0.001
Sum, mean (SD)	2.5 (1.2)	3.3 (1.5)[†]	2.2 (1.0)[£]	2.2 (1.4)[£]	<0.001

Summary statistics are expressed as total numbers and frequency distributions for categorical variables, while continuous variables are presented as mean ± standard deviation. Differences between clusters were assessed using one-way analysis of variance for continuous variables and the chi-squared test of independence for categorical variables.

[£]Significantly less prevalent compared with the rest of the cohort population.
[†]Significantly more prevalent compared with the rest of the cohort population.

Suppl. Table S6: The frequencies of comorbidities identified in each cluster from the GSCC. The columns represent the clusters, and the rows indicate the number of patients with that comorbidity and the prevalence frequency (%) of the comorbidities within that respective cluster.

**Comorbidities**	**Cluster 1:** Musculoskeletal	**Cluster 2:** Mental	**Cluster 3:** Circulatory/ Metabolic	**p-value**
N	*293*	*150*	*268*	
Anxiety, n (%)	70 (23.9)	84 (56.0) [†]	42 (15.7) [£]	<0.001
Asthma, n (%)	59 (20.1) [†]	18 (12.0)	27 (10.1) [£]	0.002
Depression, n (%)	22 (7.5) [£]	149 (99.3) [†]	16 (6.0) [£]	<0.001
Diabetes, n (%)	7 (2.4) [£]	6 (4.0)	33 (12.3) [†]	<0.001
Heart attack, n (%)	9 (3.1) [£]	12 (8.0) [£]	83 (31.0) [†]	<0.001
Heart failure, n (%)	6 (2.0)	1 (0.7) [£]	20 (7.5) [†]	<0.001
Hypertension, n (%)	32 (10.9) [£]	43 (28.7) [£]	208 (77.6) [†]	<0.001
Muscle wasting, n (%)	187 (63.8) [†]	75 (50.0)	67 (25.0) [£]	<0.001
Obesity, n (%)	4 (1.4) [£]	17 (11.3)	67 (25.0) [†]	<0.001
Osteoarthritis, n (%)	64 (21.8) [†]	10 (6.7) [£]	23 (8.6) [£]	<0.001
Osteoporosis, n (%)	98 (33.4) [†]	16 (10.7) [†]	60 (22.0)	<0.001
Peptic ulcer, n (%)	13 (4.4)	10 (6.7)	15 (5.6)	0.622
Underweight, n (%)	33 (11.3) [†]	11 (7.3)	6 (2.2) [£]	<0.001
Sum, mean (SD)	2.1 (1.3) [£]	3 (1.2) [†]	2.5 (1.2)	<0.001

Summary statistics are expressed as total numbers and frequency distributions for categorical variables, while continuous variables are presented as mean ± standard deviation. Differences between clusters were assessed using one-way analysis of variance for continuous variables and the chi-squared test of independence for categorical variables.

[£]*Significantly less prevalent compared with the rest of the cohort population.*
[†]*Significantly more prevalent compared with the rest of the cohort population.*

Section 6: Kaplan-Meier curve comparing survival in the Evaluation of COPD Longitudinally to Identify Predictive Surrogate Endpoints (ECLIPSE) cohort and the Groningen Severe COPD Cohort (GSCC)

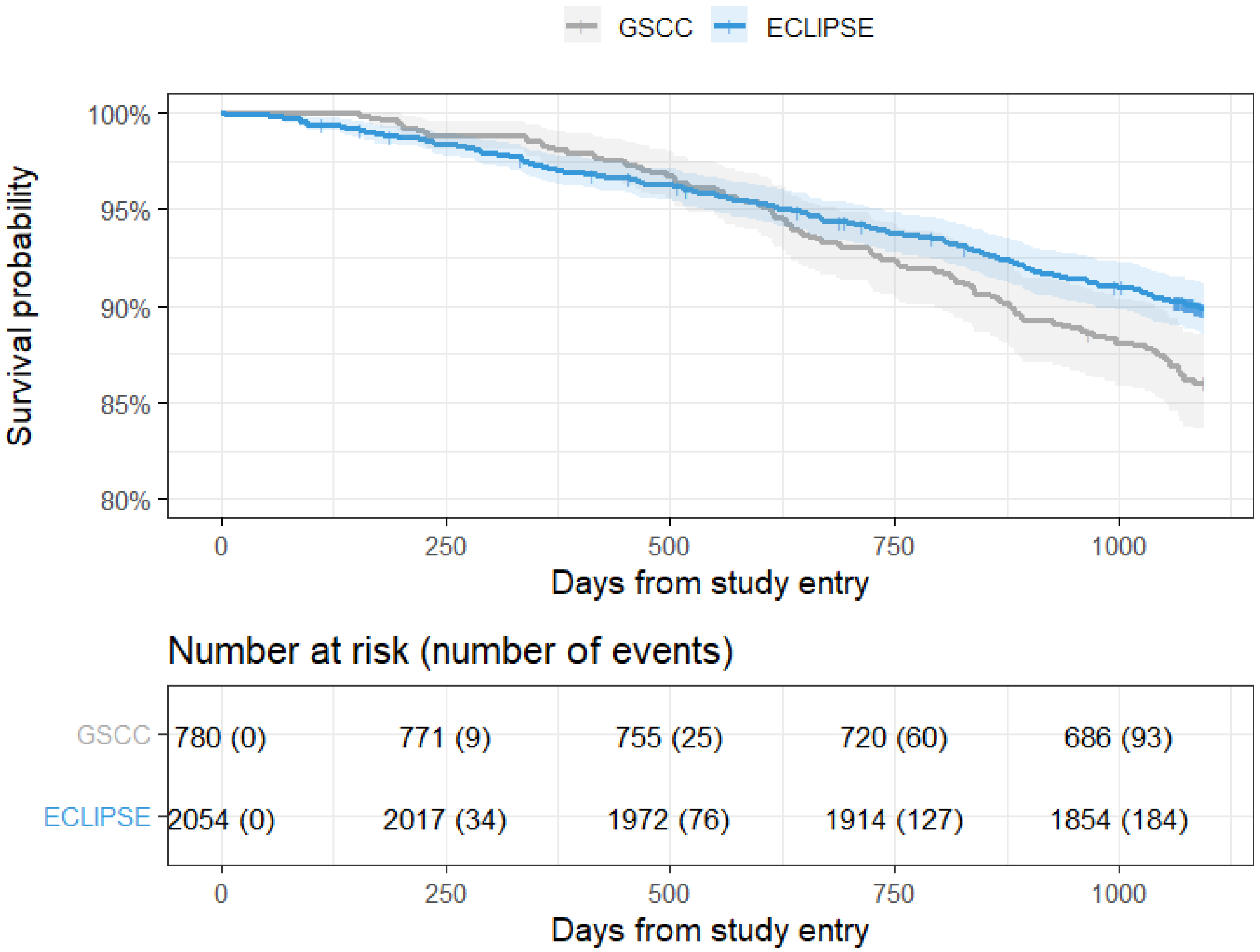

Suppl. Figure S11: Kaplan-Meier curves showing survival over 1095 days (3 years) in the ECLIPSE cohort and the GSCC.

References:

1. Wehrens R, Kruisselbrink J. Flexible self-organizing maps in kohonen 3.0. *J Stat Softw*. 2018;87(7). doi:10.18637/jss.v087.i07

2. Ward JH. Hierarchical Grouping to Optimize an Objective Function. *J Am Stat Assoc*. 1963;58(301):236-244. doi:10.1080/01621459.1963.10500845

3. Kohonen T. *MATLAB Implementations and Applications of the Self-Organizing Map (DA DOC X...)*.; 2014. http://docs.unigrafia.fi/publications/kohonen_teuvo/

4. Hamel L, Ott B. A Population Based Convergence Criterion for Self-Organizing Maps. *Proceeding 2012 Int Conf Data Min*. Published online 2012:98-104.

5. Contreras P, Murtagh F. Hierarchical clustering. *Handb Clust Anal*. 2015;(February):103-124. doi:10.1201/b19706

6. Jin Z, Shang J, Zhu Q, Ling C, Xie W, Qiang B. RFRSF: Employee Turnover Prediction Based on Random Forests and Survival Analysis. *Lect Notes Comput Sci (including Subser Lect Notes Artif Intell Lect Notes Bioinformatics)*. 2020;12343 LNCS:503-515. doi:10.1007/978-3-030-62008-0_35

7. Lundberg SM, Erion G, Chen H, et al. From local explanations to global understanding with explainable AI for trees. *Nat Mach Intell*. 2020;2(1):56-67. doi:10.1038/s42256-019-0138-9

8. Lin DY, Wei LJ, Ying Z. Checking the Cox model with cumulative sums of martingale-based residuals. *Biometrika*. 1993;80(3):557-572. doi:10.1093/biomet/80.3.557
